# Advances in Biomaterials Development and Their Energy Harvesting Application

**DOI:** 10.34133/research.1093

**Published:** 2026-03-25

**Authors:** Xue Jiang, Yong-Mei Wang, Yu-Xuan Jia, Lin-Xuan Zheng, Shun-Cheng Zhang, Ao Li, Jeong-Hyun Cho, Rusen Yang

**Affiliations:** ^1^ School of Physics, Xidian University, Xi’an 710126, China.; ^2^ Department of Electrical and Computer Engineering, University of Minnesota, Twin Cities, Minneapolis, MN 55455, USA.

## Abstract

Biomolecular self-assembly presents a convenient and promising approach for fabricating functional nanomaterials. The inherent structural and functional diversity of biomolecules facilitates the creation of a wide spectrum of biological nanomaterials through this intricate process. These versatile materials have demonstrated extensive utility in multidisciplinary domains, encompassing materials science, biomedical engineering, tissue engineering, nanotechnology, and analytical science. This review focuses on self-assembled biomolecular materials for energy harvesting applications, organized into 4 main aspects. Firstly, we introduce the self-assembly methods and key factors involved in biomolecular self-assembly mechanism, including hydrogen bonding, covalent bonds, π–π interactions, electrostatic interactions, and hydrophobic/hydrophilic interactions. Secondly, we discussed the structural characteristics of different types of self-assembled biomolecular materials and introduced their mechanical, piezoelectric, ferroelectric, semiconductor, and biocompatibility properties. Next, the application of self-assembled biomolecular materials in energy harvesting was studied, mainly focusing on piezoelectric nanogenerators, triboelectric nanogenerators, water-enabled electricity generation, and other types of energy harvesting devices. Finally, we emphasized challenges in the area encompassing not only the design of the materials and assembly regulation but also probable efforts in improving performance and application fields. We believe that this review will elucidate diverse functions of self-assembled biomaterials, paving the way for the innovative design of biomaterials not only for energy harvesting systems but also for biomaterial-based microelectronic devices.

## Introduction

The current global energy crisis and environmental pollution have become key factors hindering the improvement of human living standards. Energy, as the cornerstone of modern social development, directly impacts human quality of life and environmental sustainability through its generation, storage, and consumption. Researchers around the globe have been offering their solutions to the problem of energy shortage, and different energy harvesters have emerged to convert biomechanical, thermal, or chemical energy into electrical power. Among the energy harvesters, the most extensively studied energy harvesting devices include the piezoelectric nanogenerator (PENG), triboelectric nanogenerator (TENG), photovoltaic cell (PVC), biosolar cell (BSC), automatic wristwatch (AW), pyroelectric nanogenerator (PYENG), biofuel cell (BFC), thermoelectric generator (TEG), and endocochlear potential (EP) collector, among others [1–4].

Simultaneously, the importance of the sustainability is increasingly highlighted to ensure the survival and development of future generations. Biomaterials, as products of biological resources, characterized by renewability and biodegradability, are considered an essential component of sustainable development. Beyond their role as sustainable resources, biomaterials also embody a deeper connection to life processes through their inherent ability to form ordered structures—a phenomenon rooted in the fundamental principle of biomolecular self-assembly. Biomolecular self-assembly serves as the foundation of life, manifesting ubiquitously within living organisms. It encompasses a wide spectrum of processes, ranging from the assembly of simple lipid molecules into phospholipid bilayers [5], the folding of peptide chains into intricate protein structures [6], the formation of the DNA double helix [7], to the construction of complex organelles [8], cells [9], tissues [10], and organs [11]. These hierarchical self-assembly events involving biomolecules at various scales provide an essential framework for the functionality and activity of living organisms. The self-assembly process of biomolecules entails the spontaneous formation of ordered aggregates, guided by both dynamics and thermodynamics. This intricate process relies on the synergistic interplay of diverse noncovalent forces, including hydrogen bonding, electrostatic interactions, hydrophobic effects, π–π interactions, and van der Waals forces, which dictate the shape and functionality of the resulting assemblies [12,13]. Drawing inspiration from biological systems, utilizing biomolecules as building blocks for fabricating nanostructures with diverse size and morphology has garnered important attention and research interest. Biomolecules possess the capability to self-assemble, forming nanoscale or microscale structures, holding tremendous potential for applications in biomedicine, nanotechnology, energy, and other fields.

We present a comprehensive review of the assembly mechanisms and methods involved in the self-assembly of various biomolecules, encompassing amino acids, peptides, and proteins. The primary mechanisms driving the assembly process, such as hydrogen bonding forces, electrostatic forces, hydrophobic interactions, π–π interactions, and van der Waals forces, are discussed in detail. Additionally, we delve into the properties exhibited by the self-assembled materials, including piezoelectricity, ferroelectricity, semiconductor behavior, and biocompatibility.

Furthermore, the application of self-assembled biomaterials, particularly in the field of energy harvesting, is explored along with an overview of recent research progress in PENGs, TENGs, water-enabled electricity generation, and other types of energy harvesting devices. Finally, we address the current challenges and prospects for self-assembled biomaterials in energy harvesting. This review further provides valuable insights for readers and researchers seeking to comprehend their diverse applications, which range from energy harvesting to the development of new materials for extensive utilization in nanotechnology, materials science, analytical science, and biomedical engineering.

## A Wide Variety of Biomaterials

Biomolecules include amino acids, peptides, proteins, DNA/RNA, viruses, etc. These biomolecules are specifically chosen as focal points for investigation due to their unique combination of natural abundance, structural versatility, and inherent biocompatibility—traits that make them exceptionally well-suited for sustainable energy harvesting applications. Amino acids, for instance, exhibit remarkable chemical diversity, with side chains that can act as redox centers, enabling electron transfer crucial for energy conversion processes. Peptides, composed of amino acid chains, offer structural flexibility and can self-assemble into ordered nanostructures such as nanofibers and sheets; these architectures provide large surface areas, which enhance charge accumulation and facilitate efficient energy capture. Proteins, with their complex 3-dimensional (3D) structures, often possess inherent functionalities like piezoelectricity, allowing them to convert mechanical energy into electrical energy directly. This section mainly introduces the structure, fabrication method, and self-assembly mechanism of biomaterials.

### Amino acids

**Fig. 1.**
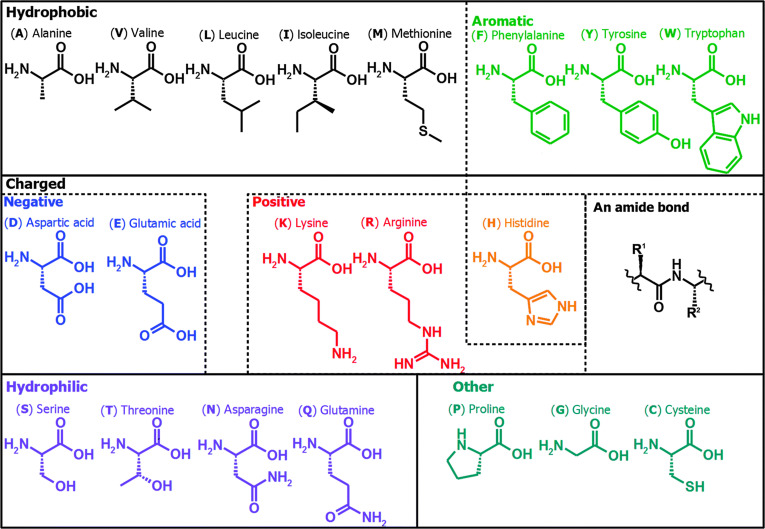
The 20 amino acids that make up the main building blocks of proteins in living organisms [161]. Adapted with permission of [161]. Copyright 2014. The Royal Society of Chemistry.

Amino acids are organic compounds containing alkaline amino groups and acidic carboxyl groups, which are the basic building blocks for peptides and proteins. Benefiting from the diversity of R-groups, a wide variety of amino acids have emerged [10]. According to the unique R-group, it can be divided into 3 categories with different hydrophobicity and other properties: aliphatic amino acids (glycine, alanine, valine, leucine, isoleucine, serine, threonine, cysteine, methionine, aspartate, glutamate, aspartame, glutamine, lysine, and arginine), aromatic amino acids (phenylalanine, tyrosine, and tryptophan), and heterocyclic amino acids (histidine and proline) (Fig. 1).

Amino acids have the following properties: (a) Chemical modification. The C terminus of amino acids is carboxyl group, and the N terminus is amino group. Due to the simple molecular structure and active site, amino acid molecules can be modified by a chemical coupling method to achieve special functions [14]. (b) Multiple stimulus responsiveness. Biological self-assembled materials are generated by the combination of amino acid/short peptide derivatives via weak intermolecular contact forces such as electrostatic interaction, hydrogen bonding, π–π stacking, and hydrophobic interaction [15]. As a result, self-assembled biomaterials are extremely sensitive to changes in their physical environment, exhibiting stimulation-response characteristic [16,17]. Changes in the environmental temperature, pH, ion type and intensity, enzymes, and light conditions induce changes in the nanostructure. (c) Chirality. Chirality is a basic property of living beings that exists widely in nature and affects the structure and properties of amino acids, short peptides, proteins, and DNA double helix. The assembled nanostructures have distinct physical and chemical characteristics due to the diversity and biocompatibility of amino acids.

### Peptides

Polypeptides are the most chemically versatile biopolymers that are made up of 20 different amino acid components. Peptide self-assemblies have found widespread applications because of their good thermostability and mechanical stability, semiconductivity, piezoelectricity, and optical characteristics. Peptides have the following properties: (a) Peptides could be easily modified at the molecular level, such as introducing substance groups and compounds (enzymes, nanoparticles, antibodies), giving peptide-based nanomaterials with tailored features. (b) Peptide self-assembly process could be well-designed by adjusting the secondary building block, such as α-helices and β-sheets [18,19]. Peptide self-assembly is dependent on the conformation of the structure, including α-helices, β-sheets, and β-hairpins. α-Helical peptide is accessible to chemical synthesis and modification, but prone to thermodynamic instability. Peptides with β-sheets can form highly ordered nanotubes, vesicles, and so on [20]. A necessary condition of β-hairpin self-assembly is the presence of bent amino acid sequence in the peptide chain segment. The efficient method to control peptide self-assembly is to control the diffusive kinetics of peptide monomers and enzymes.

### Proteins

Proteins are nature’s primary building blocks for the construction of sophisticated molecular machines and dynamic materials, which fold into unique nanometer dimensions from polypeptides. The folding of polypeptide chains introduces structural and functional complexity, inspiring scientists to design protein assemblies.

**Fig. 2.**
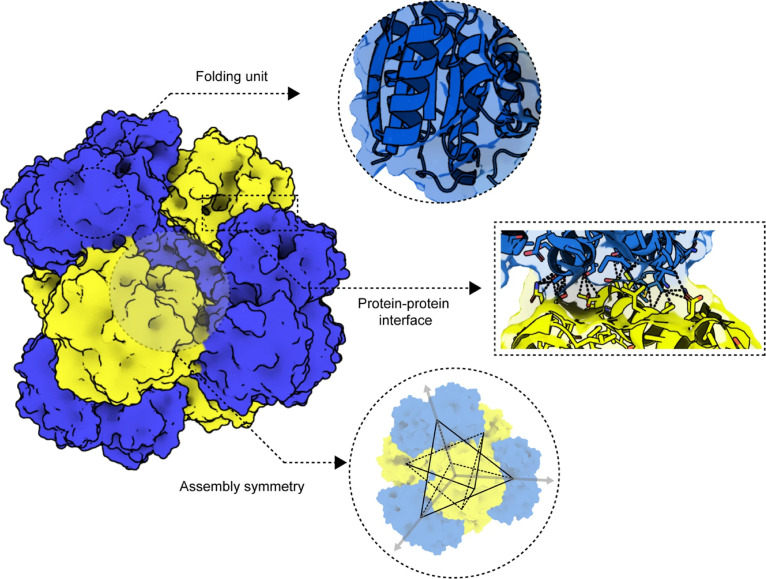
Schematic image of 3 components of supramolecular protein assembly [162]. Adapted with permission of [162]. Copyright 2014. The Royal Society of Chemistry.

It is worth noting that protein self-assembly and protein folding are 2 different processes. The polypeptide folding process is independent of environment or concentration, and it is under thermodynamic control. Meanwhile, the polypeptides are constrained to the polypeptide backbone steric/dihedral stringently. Protein assembly usually leads to 1D, 2D, and 3D ordered polymeric materials, and the connections between protein monomers are the most important determinants of the architectures, characteristics, and activities of assembled crystals. The protein self-assembly process is dependent on the environment and concentration, and is not always thermodynamic control and steric constraints [6,21]. Protein–protein interfaces, such as the helical interface, β-sheet elongation, and coordinates, determine the structure and function of protein assemblies. Assembly symmetry shows stacking order of building units and affects the physical properties of the protein assemblies (Fig. 2).

The structure of the protein assembly is essential for its property, and its practical function is often revealed during its dynamic interaction with the environment. The following properties of protein assemblies have attracted the most attention. (a) Stability. The stability of protein assemblies is important to their functions and applications; however, most natural proteins are only stable in restricted temperature and pH ranges. The targeted chemical modification and design are required to improve stability, such as decreasing the surface area of exposed solvent, increasing the amount of intermolecular interactions, and containing the conformational space of the building block. (b) Biocompatibility. Proteins are natural biomaterials that can deliver drug and recognize biological materials. (c) Mechanical properties. The protein assemblies have wonderful strength, elasticity, toughness, and self-healing properties, due to the hierarchically organized and strongly interconnected structures. By adjusting the interactions between interproteins, the mechanical characteristics can be modified extensively.

### Other biomaterials

**Fig. 3.**
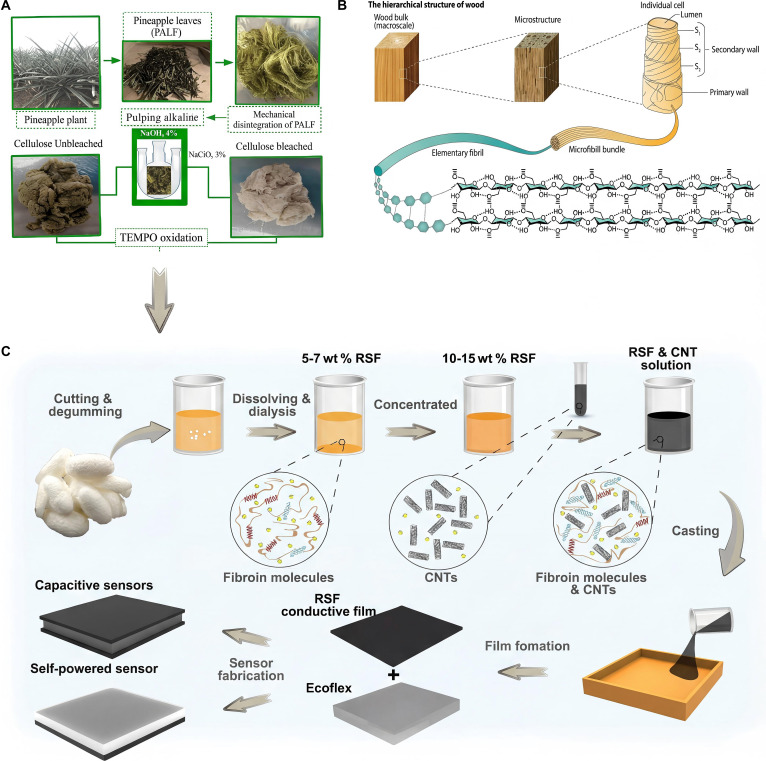
Examples of other biomaterials. Schematic of (A) cellulose [22], (B) wood [23], and (C) silk fibroin [24]. Adapted with permission of [22–24]. Copyright 2020, 2022. Springer.

In addition to the abovementioned biomaterials, there are other natural/genetically modified biomaterials, such as cellulose, wood, silk, and so on. Cellulose is composed of β-d-glucose units linked by β-1,4 glycosidic bonds. Rigid microfibrils (with a diameter of 10 to 30 nm) are formed between molecular chains through hydrogen bonds, which further assemble into a fiber network structure. The coexistence of its crystalline region (ordered arrangement) and amorphous region (disordered arrangement) endows the material with unique mechanical properties. Cellulose has a tensile strength of 300 to 700 MPa and an elastic modulus of 130 GPa, superior to most synthetic polymers. It can be gradually decomposed into glucose by cellulase and has excellent environmental compatibility and biodegradability (Fig. 3A) [22]. Wood is a lightweight and strong layered composite material formed by the interfacial self-assembly of cellulose (40% to 50%), hemicellulose (20% to 30%), and lignin (15% to 30%). The air-dried wood has a density of 0.3 to 0.9 g/cm^3^ and flexural strength of 50 to 150 MPa that is higher than many synthetic composites of the same density (Fig. 3B) [23]. Silk is mainly composed of fibroin. Its primary structure contains repeated alanine/glycine sequences that self-assemble into nanocrystalline domains through β-sheets and amorphous regions of random coils. Cocoon silk is composed of 2 fibroin fibers coated with sericin, and smooth fibers are formed after degumming. It has high mechanical toughness and biocompatibility. The tensile strength of natural silk is about 400 to 500 MPa, and the elongation at break can reach 20% to 30%, showing good combination of high strength and flexibility. Fibroin can be degraded into natural amino acids and is suitable for in vivo implant applications (Fig. 3C) [24]

## Interaction and Self-Assembly Mechanism

Although the mechanisms of biomolecular self-assemblies are complicated, the basic interactions of biomolecular self-assemblies have been well expounded with the hydrogen bond, covalent bond, π–π interaction, electrostatic interaction, and hydrophobic/ hydrophilic interactions [12,25].

### Hydrogen bond

Hydrogen bonding is a special intermolecular or intramolecular interaction between hydrogen atoms and atoms with large electronegativity. Intermolecular hydrogen bonding interactions play an important role as a more common driving force in the self-assembly of biomolecules. Intermolecular hydrogen bonding interactions can promote the orderly assembly of biomolecules and strengthen the connection between biomolecules, thus forming biomolecule-based crystals with diverse functions [12,26].

**Fig. 4.**
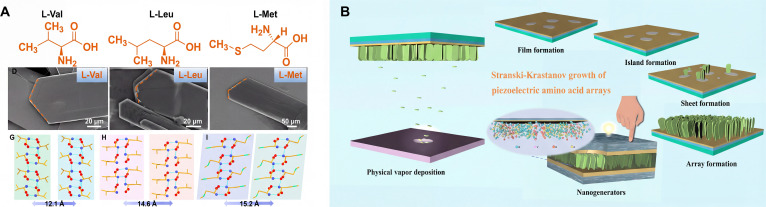
(A) Self-assembly growth of amino acids through hydrogen bond interactions [27]. Adapted with permission of [27]. Copyright 2023 AAAS. (B) Schematic illustration of the Stranski–Krastanov growth mechanism of self-assembled arrays [28]. Adapted with permission of [28]. Copyright 2022 American Chemical Society.

The hydrogen bond interaction between amino and carboxyl groups contributes greatly to the stability and functionality of the amino self-assembly structure. The weak interaction between oxygen (O) and hydrogen (H) atoms results in low mechanical strength, allowing for marked ion displacement under stress, thereby favoring a strong piezoelectric response. The hydrogen bonds in biological crystals are closely related to their physical properties, and these crystals have the potential to serve as functional biomaterials for high-performance energy harvesting devices. Our group has employed a hydrogen bonding network in double-layer amino acid crystals to develop a strategy for enhancing physical properties (Fig. 4A) [27]. The interatomic interactions and density of state (DOS) properties were explored by density functional theory (DFT) calculations. We grew vertical arrays of amino acids, valine, leucine, and isoleucine by physical vapor deposition (PVD) processes and revealed their Stranski–Krastanov growth mechanism based on intermolecular hydrogen bonding interactions, and these arrays showed substantially enhanced piezoelectric properties (Fig. 4B) [28].

**Fig. 5.**
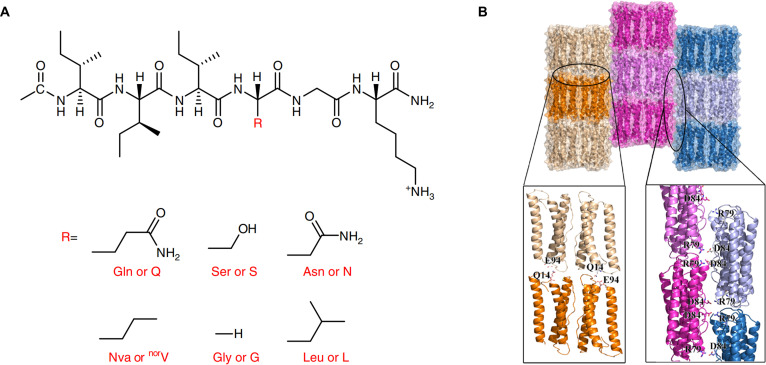
(A) Preparation of distinctive nanoribbons through the interactions of side-chain hydrogen bonding among β-sheets [30]. Adapted with permission of [30]. Copyright 2018 Springer. (B) Formation of HuHF1_−134_ nanotube arrays through hydrogen bond interaction in the crystal [32]. Adapted with permission of [32]. Copyright 2018 American Chemical Society.

Wang and colleagues [29] synthesized D-J-1 (d-Pro-d-Phe-d-Lys-d-Leu-d-Ser-d-Leu-d-His-d-Leu-NH_2_), which was an all-d-enantiomer of peptide Jelleine-1 (J-1). The hydrogen bonding interaction during the formation of D-J-1 hydrogel was evaluated by ThT (thioflavin T) binding assay. As a benzothiazole salt fluorescent probe, ThT can visualize and quantify the presence of β-sheet conformation, which is mainly driven by hydrogen bonding, thus indirectly confirming the involvement of hydrogen bonding in the formation of D-J-1 hydrogel. Xu and colleagues [30] designed peptides based on the general formula Ac-I 3 XGK-NH_2_. The side-chain hydrogen bonding (H-bonding) interaction among β-sheets (defined as polar zippers) led to distinctive nanoribbons, while the insertion of hydrophobic amino acid residues resulted in amyloid-like nanofibrils (Fig. 5A) [30]. Gazit and colleagues [31] confirmed that tryptophan assembles into dipeptides via hydrogen bonding interactions by cyclo-phenylalaninetryptophan (cyclo-FW) and cyclo-tryptophan-tryptophan (cyclo-WW) crystals data and MD (molecular dynamics) simulations, demonstrated that tryptophan-based aromatic dipeptide supramolecular structures are direct wide-gap semiconductor, and pointed out that hydrogen bonds and aromatic interactions endow aromatic dipeptide supramolecular structures with unique properties such as rigidity and thermal stability, enabling them to serve as bio-organic supramolecular semiconductors for the fabrication of biocompatible microelectronics to realize applications like energy harvesting.

Hydrogen bonds are also important in protein assemblies. Wang et al. [32] developed a novel strategy to convert the 24-mer cage-like ferritin into non-native 8-mer protein nanorings by selectively eliminating a key subunit interface (the C_3_–C_4_ interface) through truncation of the inherent C terminus. Crystallographic analysis revealed that these nanorings self-assemble into nanotubes via head-to-tail hydrogen bonding, with adjacent nanotubes staggering to form 3D porous architectures containing round pores (8.04 Å^2^) and 4-angle star-shaped pores (10.54 Å^2^) (Fig. 5B). Ouyang and colleagues [33] proposed a protein-directed assembly strategy that used hydrogen bonding to organize proteins and organic linkers into a highly crystalline hybrid framework. The Fourier transform infrared (FTIR) measurement and theoretical modeling suggested that protein surface residues and organic linkers were connected with hydrogen bonds during the assembly process.

### Covalent bonds

**Fig. 6.**
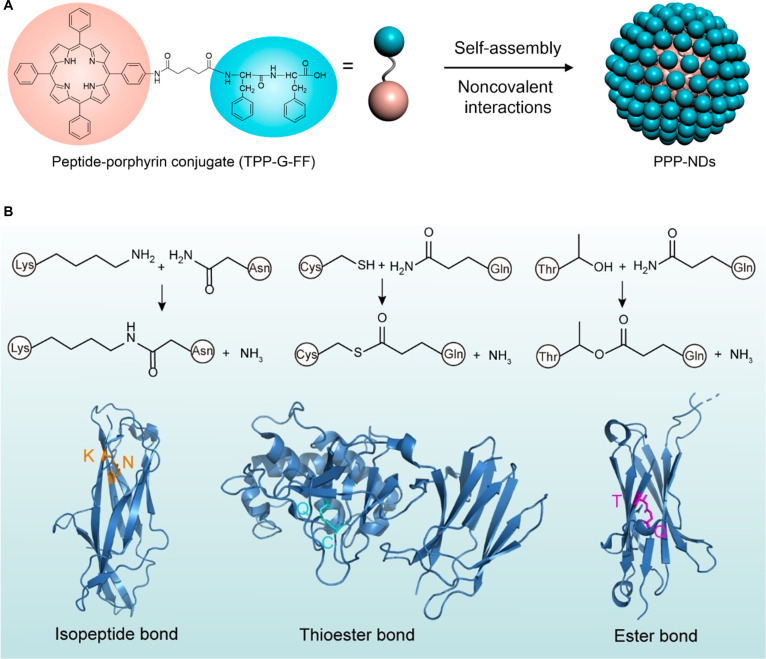
(A) Schematic diagram of chromopeptide formation through covalent coupling [34]. Adapted with permission of [34]. Copyright 2017 American Chemical Society. (B) Intermolecular covalent crosslinks in Gram-positive bacteria surface proteins [35]. Adapted with permission of [35]. Copyright 2022 Welly.

Although the self-assembly of biomaterials relies mainly on noncovalent interactions, relatively weak covalent bonds such as coordination bonds may also be involved. These interactions work cooperatively, leading to complex yet coherent self-assembly processes and well-defined architectures. For example, the peptide porphyrin chromopeptide (TPP-G-FF) was designed by Yan and colleagues [34] through the covalent conjugation of FF with porphyrin, a photosensitive pigment molecule. The MD simulation showed that the combined effects of π-stacking and hydrophilic interactions contribute to the stabilization and organization of chromopeptide assemblies, thereby facilitating the formation of well-defined nanodots (Fig. 6A). Additionally, surface proteins of Gram-positive bacteria have enhanced mechanical stability through 3 intramolecular covalent bonds: isopeptide bond (Lys–Asn/Asp), thioester bond (Cys–Gln), and ester bond (Thr–Gln), to resist physical stress and enzymatic degradation in the host environment. These bonds form spontaneously during protein folding. The response characteristics were revealed by atomic force microscopy (AFM) single-molecule force spectroscopy. The isopeptide bond can withstand a breaking force of 1.5 nN, and the thioester bond irreversibly binds to the host ligand under a force above 35 pN (Fig. 6B) [35]. A lignin-based macromolecular autocatalytic system was used by Nie and colleagues [36] to initiate the rapid in situ polymerization of monomers into polyacrylamide (PAM) chains. DFT and ^1^H nuclear magnetic resonance (NMR) results indicate that covalent-like hydrogen bonds are formed between Tr molecules and PAM chains; additionally, cellulose nanofibers (CNFs) were incorporated as a gel network enhancer. Through this approach, a hydrogel-based triboelectric material was successfully prepared, featuring versatile characteristics such as 2,840% stretchability, antifreeze performance at −18 °C, and antidehydration ability at 40 °C.

### π–π interaction

Some biomolecules, including peptides, proteins, DNA, enzymes, and viruses, contain aromatic motifs, which make it possible to form highly ordered superstructures by π–π interactions [12]. Amino acids with nonpolar side chains can form nanostructures through π–π interactions. Das and colleagues [37] constructed a carboxybenzyl-protected, l-phenylalanine-appended bola-amphiphile. NDI-1 and the concentration-dependent ^1^H NMR proved the existence of π–π interaction between the NDI chromophores. π–π interaction can promote peptide self-assembly, particularly for π-conjugated peptides, like aromatic peptides.

**Fig. 7.**
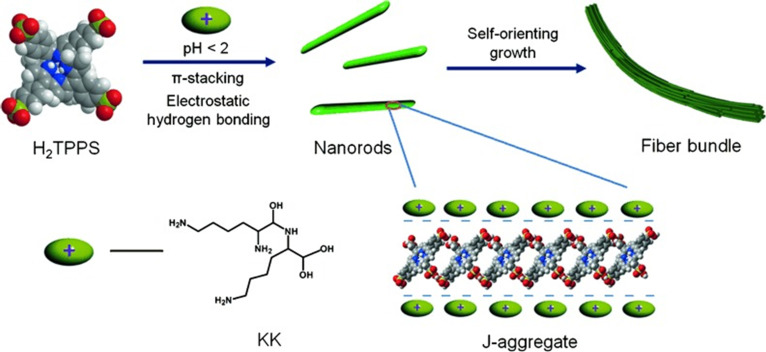
Schematic diagram of the self-assembly of nanorods and their aggregation into fibrous bundles [38]. Adapted with permission of [38]. Copyright 2015 Wiley.

In pure organic solvents like toluene, the solvent effect might make the π–π interaction more prominent. Yan and colleagues [38] co-assembled l-Lys-l-Lys and porphyrins to form dipeptide–porphyrin nanorods with good photostability and sustained photocatalytic activity. The self-assembly process was driven by electrostatic and hydrogen bonding between dipeptide–porphyrin molecules and π–π interactions between porphyrin–porphyrin molecules, which was proved by ultraviolet/visible (UV/Vis) absorption spectrum and FTIR spectrum. The nanorods further aggregated into a fiber bundle structure under van der Waals forces (Fig. 7). Tao et al. [31] examined peptide–protein interactions in the assembly process of peptides and graphene oxide (GO) with pyrene that served as a label of peptides and enhanced the interaction between the peptide and GO with π–π interactions and hydrophobic interactions. π–π stacking interactions enabled the ordered protein self-assembly and nanoribbons by introducing histidine residues into RhuA (l-rhamnulose-1-phosphate aldolase) [39]. In addition, Raman data confirmed the presence of cation–π and π–π interactions between imidazole (IM) rings and carbon nanotube (CNT) surfaces in IM-cured composites, as reported by Mandal et al. [40]. The interfacial adhesion between the filler and the elastomer is markedly improved, which was used in dynamically self-healable, stretchable piezoresistive sensors and TENGs.

### Electrostatic interaction

The electrostatic interaction between positive and negative charges does not involve electron sharing or transfer and plays a crucial role in the self-assembly of peptides, proteins, enzymes, etc., leading to the formation of nanostructures [12,41]. Based on the results of the characteristic distances predicted by geometry optimization being consistent with x-ray scattering data and transmission electron microscopy observations, Boulmedais and colleagues [42] proposed that core–shell cylinders are formed in which polycation chains decorate the micellar structures of Fmoc-FFpY peptides through electrostatic interactions between the charged amine groups of the polycations and the phosphate groups of the peptides. The positively charged amine groups of poly(allylamine hydrochloride) (PAH) electrostatically attract the negatively charged phosphate groups on Fmoc-FFpY, triggering instantaneous gelation without peptide dephosphorylation. This interaction stabilizes a core–shell cylindrical structure, where PAH chains coat the surface of Fmoc-FFpY micelles, enhancing mechanical properties.

**Fig. 8.**
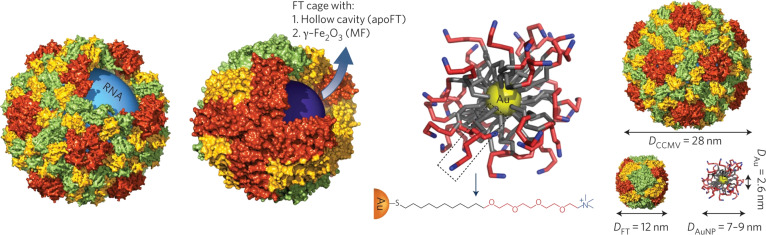
Self-assembly of binary protein cage-nanoparticle superlattice materials by electrostatic interaction [44]. Adapted with permission of [44]. Copyright 2015 Springer.

The electrostatic interaction can greatly affect protein self-assembly, because proteins are highly charged [43]. Kostiainen et al. [44] found that the lattice parameters obtained from small-angle x-ray scattering (SAXS) fitting are consistent with the electron density map obtained by cryogenic electron tomography (cryo-ET), providing structural-level evidence that negatively charged protein cages (e.g., cowpea chlorotic mottle virus and ferritin) can electrostatically interact with positively charged gold nanoparticles, guiding their self-assembly into 3D superlattices. Such electrostatically driven self-assembly not only enables the creation of novel superlattices with potential in magnetic resonance imaging but also provides a biocompatible platform for developing nanomaterials in biological applications. Ahn et al. [45] oxidized wCF to wCF-COOH using piranha solution and then coupled aniline through electrostatic interaction and in situ oxidative polymerization to obtain the carbon fiber–polyaniline emeraldine salt (wCF.PANI.ES) composite material. This composite was used as one of the triboelectric layers in the contact-separation mode TENG, which generates charges by harvesting energy together with a polyvinylidene difluoride (PVDF) membrane (Fig. 8).

### Hydrophobic/hydrophilic interaction

**Fig. 9.**
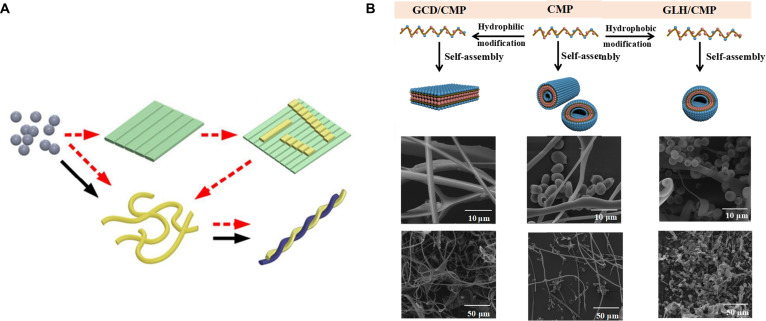
(A) Schematic diagram of self-assembly pathways of different nanostructures [47]. Adapted with permission of [47]. Copyright 2016 RSC. (B) Microtopography properties of CMP self-assemblies controlled by hydrophilic and hydrophobic interactions between CMP [48]. Adapted with permission of [48]. Copyright 2021 Springer.

Within living organisms, numerous biomolecules possess both hydrophilic and hydrophobic properties, enabling them to self-assemble and form specific structures, such as phospholipid membranes [46]. Amino acids, peptides, and proteins can also form highly ordered structures due to their hydrophilic–hydrophobic properties. Chen and colleagues [47] proposed 2 distinct assembly pathways (in solution and on the substrate) for the self-assembly of small peptide amphiphile (NapFFKYp). The self-assembly process was initiated from peptide aggregates and followed by the formation of nanofibers in solution. The nanofibers were then twisted to yield highly ordered structures. Meanwhile, the peptide molecules self-assembled on the substrate to form nanosheets that could be converted into nanofibers by water molecules (Fig. 9A). MD simulations supported the presence and great influence of hydrophobic interaction during the formation of nanofibers. Liu and colleagues [48] investigated the grafting of collagen mimetic peptide (CMP) with both hydrophilic and hydrophobic groups, and used FTIR to analyze shifts in peak positions and intensity variations of CMP’s characteristic functional groups pre- and post-modification, revealing that hydrophilic interactions promote fiber aggregate formation, while hydrophobic interactions favor microsphere formation (Fig. 9B). This study presents a comprehensive analysis of the impact of hydrophilic and hydrophobic forces on the assembly kinetics, as well as the morphological and structural characteristics of self-assembled CMP biomacromolecules. Du and colleagues [49] bound cationic polyethylenimine (PEI) molecules to the surface of cellulose fibers during the pulping process, enhancing the interfacial hydrophilic interactions between fibers. This substantially improved the wet strength of the cellulose paper as well as its triboelectric properties.

### Synergistic interactions

The self-assembly of biomolecules is a sophisticated process orchestrated by the synergistic and competitive interplay of multiple noncovalent forces, including hydrophobic interactions, hydrogen bonding, electrostatic forces, and van der Waals interactions. These forces do not act in isolation; rather, they form a dynamic, context-dependent network that guides the pathway and determines the final structure [50,51]. For instance, hydrophobic effects often provide the initial driving force for aggregation, while hydrogen bonding confers structural specificity and directionality. The dominance of a particular force is highly sensitive to environmental conditions such as pH, ionic strength, and temperature. A change in pH can alter charge distributions, thereby shifting the balance between electrostatic attraction and repulsion, which in turn can amplify or suppress the role of hydrophobic forces. Understanding this intricate hierarchy and cooperativity is not merely an academic exercise—it is the foundational knowledge required to achieve precise spatiotemporal control over self-assembly, enabling the rational design of complex and functional biomaterials from the bottom up.

## Physical and Chemical Properties of Self-Assembled Biomolecular Materials

### Mechanical property

The mechanical properties of biomaterials, including elastic modulus, viscoelasticity, hardness, toughness, and stretchability, are of great importance in the fundamental studies and applications [52]. The elastic modulus of biomaterials is usually low and matches the soft tissues of the human body [53]. Many extracellular matrix (ECM)-based natural materials derived from natural tissues, such as collagen and glycosaminoglycans (GAGs), have near physiological level elasticity. For example, collagen shows elastic moduli of 1 to 10 GPa [54] and traditional inorganic materials have elastic moduli of 10 to 10^2^ GPa [55]. To manipulate the elasticity of these materials, different strategies have been developed. A common way to achieve elastic regulation is to change the molecular composition of a material. For example, the elasticity of composite hydrogels was effectively modulated by simply changing the molecular weight of HA (hyaluronic acid) molecules, leading to hydrogels of enhanced stiffness without compromising the biological activity of HA [56].

Viscoelasticity is a crucial mechanical characteristic found in natural ECM-derived materials like type I collagen gels and various tissues such as muscle, brain, and adipose [57]. Liu and colleagues [58] developed a series of injectable, viscoelastic PEGylated poly (glycerol sebacate) (PEGS-OH) hydrogels that are chemically cross-linked by clicking, and they investigated the role of viscoelasticity in the regeneration of developmental tissues. Eighty percent crosslinked PEGS-OH with incomplete network slip promoted a marked increase in adhesion and differentiation of bone mesenchymal stem cells (BMSCs) compared to 100% and 60% crosslinking degrees.

Hardness is the ability of a material to resist local compression. The hardness of biomaterials is usually lower in order to better fit the soft tissues of the human body. Wu and colleagues [59] subjected the silica-based natural sponge skeleton to low-temperature hydrothermal mineralization treatment in calcium hydroxide solution. Calcium hydrated silicate was grown in situ on the surface of the silica matrix, achieving a tight bond between the bioactive layer and the matrix, and finally, a naturally derived bioceramic scaffold was obtained. The naturally derived bioceramic scaffold retains the flexibility and resilience of the original sponge skeleton, can be arbitrarily cut and tailored into specific shapes, and has ideal processing performance, meeting the repair needs of thin-walled bone tissues of different shapes. Without the introduction of polymer components, the naturally derived flexible bioceramics possess flexural strength and fracture toughness far higher than those of traditional bioceramics and bioceramic-based composite scaffolds, featuring combined mechanical properties of light weight and high strength.

Biological materials usually have a high toughness to ensure that they do not break easily when impacted. For instance, the fracture toughness of vertical bone can reach up to 12 MPa m^−2^ [60], while most structural ceramics have a toughness of roughly 1 to 3 MPa m^−2^ [61]. Bin and colleagues [62] selected eco-friendly natural rubber (NR) as the “toughening agent” for buckypaper (BP). Through a simple vacuum filtration method, NR-toughened BP (abbreviated as NR-BP) was prepared. The NR-BP combines flexibility and high electrical conductivity, expanding its applications in fields such as electromagnetic shielding, thermal conductivity, Joule heating, and TENGs, providing a new strategy for the preparation of multifunctional wearable electronic devices.

Stretchability is an essential property for wearable devices to match varying strains when interfacing with soft tissues or organs. While piezoelectricity has broad application potentials as tactile sensors, artificial skins, or nanogenerators, enabling tissue-comparable stretchability is a main roadblock due to the intrinsic rigidity and hardness of the crystalline phase. Mao and colleagues [63] reported an amino acid-based piezoelectric biocrystal thin film that offers tissue-compatible omnidirectional stretchability with unimpaired piezoelectricity. Built on this structure, a tissue-compatible stretchable PENG was developed, which could conform to various tissue surfaces, and exhibited stable functions under multidimensional large strains. Yu and colleagues [64] fabricated cyanoethyl substituted 1.5 hydroxyls on cellulose’s d-glucose, turning its chain into a coiled structure, creating petrochemical-free S-ionogel. It has ~1,000% elongation and good biocompatibility and aids self-powered e-skins.

### Piezoelectricity

The piezoelectric effect refers to the internal polarization of a material when it is subjected to an external force, resulting in the appearance of equivalent positive and negative charges on opposite surfaces [65]. The ordered structure, low symmetry, and absence of inversion centers in biomaterials typically render them piezoelectric properties [66]. The amino acid glycine, which is the simplest in structure, can be assembled into 3 crystal types: α, β, and γ [67]. Among them, the β-glycine and γ-glycine exhibit a polar noncentrosymmetric arrangement, whereas the α-glycine crystallization demonstrates centrosymmetry. Consequently, the β and γ phases of glycine display piezoelectric behavior, while the α phase of glycine exhibits nonpiezoelectricity [68]. The formation of peptide nanotubes (PNTs) involves the self-assembly of numerous organic molecules, resulting in the creation of a group of organic nanotubes [69]. The diphenylalanine (FF) PNTs possess a noncentrosymmetric hexagonal crystal structure devoid of a reversal center, thereby exhibiting piezoelectric properties [70], and *d*_33_ was estimated in the range of 5 to 50 pm/V [71,72]. Due to the synergy of low permittivity (*ɛ*_33_), low elastic constant (*c*_33_), and dominant shear piezoelectric coefficients (*d*_33_), the FF peptide nanowires demonstrate superior piezoelectric properties (*d*_33_ = 18 pm/V, *ɛ*_33_ = 4, *c*_33_ = 24 GPa), generating markedly higher voltages compared to zinc oxide (*d*_33_ = 11.7 pm/V, *ɛ*_33_ = 12, *c*_33_ = 211 GPa), lead zirconate titanate (*d*_33_ = 152 pm/V, *ɛ*_33_ = 450, *c*_33_ = 113 GPa), and barium titanate nanowires under the same applied forces [71]. It is worth noting that the piezoelectric coefficients of most biomaterials are lower than those of traditional inorganic materials. For example, the *d*_33_ values of amino acids range from approximately 5 to 178 pC/N, peptides from 13.8 to 73 pC/N, proteins from 1 to 38 pC/N, and viruses from 0.77 to 13.2 pC/N [73]. In contrast, the piezoelectric coefficient (*d*_33_) of perovskite ceramics, a typical inorganic material, can be as high as 200 to 3,000 pC/N [74]. However, the inherent flexibility and biocompatibility of biomaterials are crucial for wearable/implantable devices. For example, natural protein fibers like silk [75] and spider silk [76] possess both piezoelectricity and biocompatibility, making them suitable for application in wearable/implantable devices.

### Ferroelectricity

Ferroelectricity is a property exhibited by certain materials that can undergo spontaneous polarization, which can be reversibly altered in the presence of an applied electric field. Some biomolecules, such as β-glycine and γ-glycine [77], demonstrate ferroelectric behavior; however, β-glycine exhibits greater susceptibility to polarization conversion than γ-glycine and therefore possesses superior ferroelectric properties. The self-assembled FF nanotubes were also observed to exhibit ferroelectric behavior because of dipole alignment [78]. Nanocellulose films demonstrate ferroelectric properties when subjected to high electric fields. Additionally, it has been discovered that all nucleobases in DNA and RNA exhibit zero-field polarization and square hysteresis curves, displaying a butterfly-like characteristic of the total energy with respect to the electric field [79]. This suggests the potential presence of ferroelectricity in these biological materials.

### Semiconductor properties

Semiconductors are a class of materials with certain energy band structures and exhibiting electrical conductivity between conductors and insulators, and they play a crucial role in the electronics industry as essential components and integrated circuits. The semiconductor properties of self-assembled biomaterials are determined by their molecular structure and assembly mode, making them highly versatile for applications in the fields of optics and electronics. For instance, it can be utilized for the fabrication of organic transistors, which possess the benefits of cost-effectiveness and facile production. Among them, pigment peptides exhibit a relatively uncomplicated molecular structure, flexible and adjustable photophysical properties, as well as programmable self-assembly capabilities, thereby showcasing the astute amalgamation of optical and biological functionalities [80]. Furthermore, self-assembled biomaterials can also be utilized for the production of optoelectronic devices, including integrated optical devices and organic light-emitting diodes (LEDs) [81]. The versatile nature of these materials enables their wide-ranging applications in the fields of electronics, optics, and medicine.

### Biocompatibility

The biocompatibility of a material often refers to its ability to effectively interact with biological tissues and organs while minimizing immune or inflammatory responses within acceptable limits. It is widely acknowledged that substances closely resembling natural tissues are more readily assimilated by organisms. Biomolecules offer a promising avenue for addressing biocompatibility challenges [82]. Material performance is intricately linked to composition and structure, mirroring the multi-level assembly observed in natural tissues through molecular self-recognition. The development of novel biomedical materials featuring biomimetic tissue structures represents a prominent frontier in current life science research. For instance, the biocompatibility of TENG based on microbial cellulose (BC) is reflected in aspects such as cytotoxicity, blood compatibility, degradability, and antibacterial properties [83].

## Energy Harvesting Based on Biomaterials

### Energy harvesting based on the piezoelectric effect

#### Introduction of PENGs

PENGs employ the piezoelectric effect to harvest energy from the surrounding environment and convert it into electrical energy, which can be used to construct self-powered systems for maintenance-free and sustainable operation [84]. A key advantage of PENGs lies in their ability to efficiently tap into abundant mechanical energy sources. Mechanical energy is widely available in nature, and many different forms of mechanical energy are generated by human activity alone, such as walking, running, blood circulation, and heartbeat. Biomechanical energy can be converted into electrical energy by PENGs to power low-power electronic devices [65]. Today, implantable medical devices such as pacemakers, defibrillators, and brain stimulators rely on conventional batteries whose capacity, size, and lifespan limit the application of these devices [85]. The emergence of PENGs offers a good solution to these problems. Many types of PENGs have been introduced based on ZnO nanowires [86,87], GaN nanowires [88,89], PVDF [90], and poly(vinylidenefluoride-co-trifluoroethylene) [P(VDF-TrFE)] [91,92]. However, few of these PENGs have both high electrical output characteristics and flexibility, and their biocompatibility needs further investigation.

#### Biomaterial-based PENGs and application

**Fig. 10.**
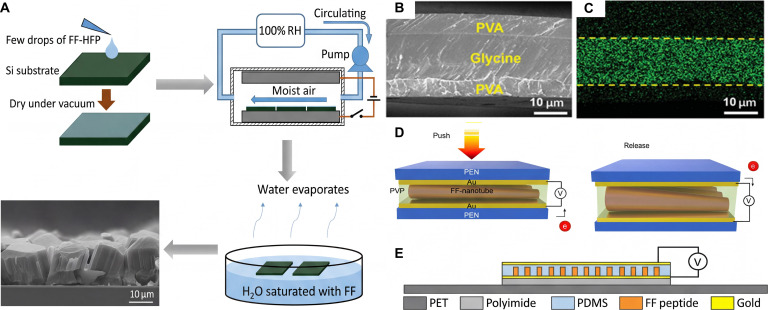
Biocompatible nanogenerator based on different biomolecules. (A) Schematic illustration of the fabrication process of FF peptide microrods [95]. Adapted with permission of [95]. Copyright 2015 Elsevier. (B) Cross-sectional scanning electron microscopy (SEM) image of a sandwich-structured film [98]. Adapted with permission of [98]. Copyright 2021 AAAS. (C) Corresponding energy-dispersive spectroscopy (EDS) map of N, confirming that the center layer is glycine [98]. Adapted with permission of [98]. Copyright 2021 AAAS. (D) Schematic illustration of FF nanotube piezoelectric energy harvester [70]. Adapted with permission of [70]. Copyright 2018 American Chemical Society. (E) Schematic of the FF peptide nanogenerator attached to a PET beam [71]. Adapted with permission of [71]. Copyright 2018 Elsevier.

In recent years, piezoelectric biomaterials have been widely studied because they possess a high degree of homogeneity [73,93,94], good biocompatibility, and ease of degradation. Some studies have found that the materials used to make PENGs can be derived from amino acids, peptides, etc. Yang and colleagues [95] fabricated vertically aligned diphenylalanine (FF) microrods on various substrates via an epitaxial growth method for the first time and measured an effective piezoelectric coefficient *d*_33_ of 9.9 pm/V from the solid single crystal (Fig. 10A). Then, they further achieved control of polarization with an electric field applied during the peptide self-assembly process, enhanced the effective piezoelectric constant *d*_33_ to 17.9 pm/V [96], and constructed a PENG based on this, which produced an open-circuit voltage of 1.4 V and a power density of 3.3 nW cm^−2^.

Amino acid, as an important branch of biomaterials, exhibits promising piezoelectric properties. Iitaka [97] discovered that γ-glycine has piezoelectricity and excellent thermal stability, and Guerin et al. [68] used DFT to find that both γ-glycine and β-glycine have high piezoelectric voltage constants, with the values reaching 0.46 and 8.13 V mN^−1^, comparable to piezoelectric ceramics like lead zirconate titanate (PZT). However, glycine crystals are brittle, which would limit their application in flexible devices. Yang et al. [98] fabricated a piezoelectric glycine film with polyvinyl alcohol (PVA)–glycine–PVA sandwich structure (Fig. 10B and C), which showed markedly improved mechanical flexibility. The film has a piezoelectric coefficient of 157.5 × 10^−3^ V mN^−1^, which is comparable to commercially available piezoelectric polymers like PVDF. Besides, the piezoelectric glycine–PVA film is water-soluble and dissolves into an aqueous solution in a short period, which shows great potential in the field of transient implantable electromechanical devices.

The molecular structure influences the electronic and supramolecular structure of biomolecular assemblies, leading to remarkable changes in piezoelectric response. Cai and colleagues [71,99] explored the potential of supramolecular engineering to enhance the piezoelectricity of amino acid-based assemblies. The study revealed that simple variations in the acetylation of amino acid side chains resulted in an increased polarization of supramolecular arrangements, leading to a marked enhancement in piezoelectric response. Additionally, the acetylation chemical modification imparted a higher maximum piezoelectric tensor compared to most naturally occurring amino acid assemblies. The predicted maximum piezoelectric strain constant and piezoelectric voltage constant of acetylated tryptophan (L-AcW) components reached 47 pm/V and 1,719 mV m/N, respectively, comparable to common inorganic materials such as bismuth triborate crystals. This work demonstrated the power output of amino acid-based PENGs through the illumination of LEDs, showcasing the systematic tuning of piezoelectric response in amino acid-based components through supramolecular engineering, facilitating the development of high-performance functional biomaterials from simple, readily available, and customizable building blocks.

Beyond single amino acids, polypeptide self-assemblies also exhibit excellent piezoelectric properties. Kholkin et al. [72] discovered that self-assembled FF PNTs exhibit strong shear piezoelectric activity. This discovery opened a broad path for a new generation of biocompatible piezoelectric devices. Lee et al. [70] developed large-scale unidirectionally polarized, neatly aligned FF PNTs and fabricated a peptide-based piezoelectric energy harvester (Fig. 10D). The electricity generated by this energy harvester can power multiple liquid crystal displays (LCDs) when an external force is applied, and this result contributes to the application of peptide-based energy harvesting devices in the biomedical field. Jenkins et al. [71] developed a complete 2D model of a flexible PENG based on FF peptide microrods and fabricated a flexible PENG based on a vertical array of FF peptide microrods (Fig. 10E). The model was successfully validated by characterizing the piezoelectric response of the PENG, and finite element modeling was applied in the design, optimization, and study of FF peptide-based PENGs. Furthermore, biomolecules such as silk proteins [100], lysozyme [101], thymidine [102], and phage [103] also have the potential to be used in the manufacture of PENGs.

PENGs with biocompatibility and even biodegradability [104] have been explored for biomedical purposes and have played an important role in the development of implantable systems, such as powering implantable devices [105,106], recording biological signals, and stimulating muscles and the nervous system in therapy. However, the study of degradable PENGs is still limited. Poly-l-lactic acid (PLLA) is a crystalline polymer known for its excellent biodegradability and biocompatibility, making it widely used in implants approved by the Food and Drug Administration (FDA) for food and pharmaceutical applications. The thermodynamically stable conformation of PLLA is the α-crystalline structure, where the introduced dipoles of carbonyl (C=O) groups do not align along the main chain of the polymer. However, under appropriate processing, it exhibits piezoelectric characteristics, such as electrospinning, which allows the alignment of dipoles in a unidirectional manner along the stretching direction, forming a β-crystalline structure with a piezoelectric coefficient *d*_14_ of 12 pC N^−1^ [107].

**Fig. 11.**
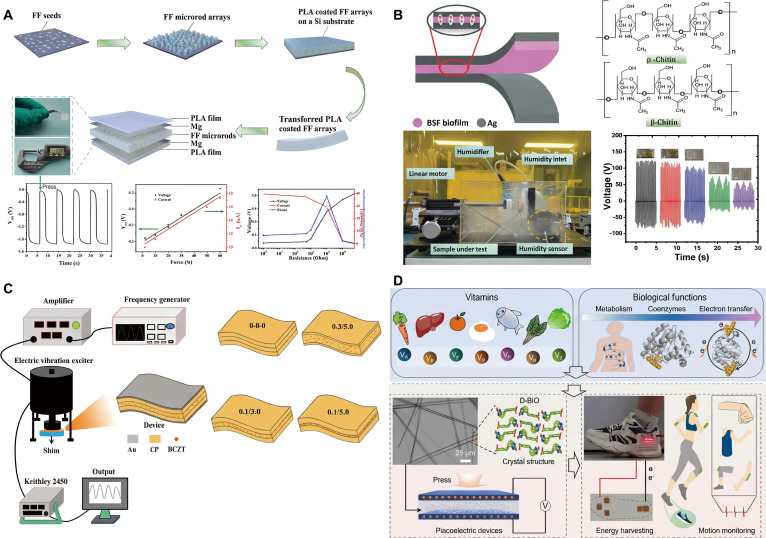
(A) Schematic diagram of the fabrication of degradable PENGs [108]. Adapted with permission of [108]. Copyright 2021 Elsevier. (B) PENG’s device structure and possible mechanism, and effect of different relative humidity levels on the BSF-TENG device [109]. Adapted with permission of [109]. Copyright 2024 Wiley. (C) Sketch of measuring the output performance of PENGs and the comparison of the output performance between the 1/3-0-1/3 PENG and some typical cellulose-based PENGs [110]. Adapted with permission of [110]. Copyright 2024 RSC. (D) Natural sources and biological functions of vitamin molecules. D-BIO assemblies with higher piezoelectricity were used to fabricate PENGs for energy harvesting and human motion monitoring among vitamin-based self-assemblies [111]. Adapted with permission of [111]. Copyright 2025 Wiley.

Our group reported a novel degradable PENG employing a free-standing polylactic acid (PLA) film embedded with arrays of diphenylalanine microrods. This design effectively enables the stiff PLLA polymer to detach the rigid microrods from the silicon substrate and uniformly distribute external forces for efficient energy conversion. The PENG demonstrates outstanding performance, generating a maximum output voltage of 1.78 V and achieving a power density of 1.56 W m^−3^. Moreover, the device exhibits complete dissolution within 25 d at 60 °C in alkaline, acidic, and phosphate-buffered saline solutions. This remarkable degradability of the PENG offers a practical solution for powering transient electronics while simultaneously reducing the environmental impact (Fig. 11A) [108].

In addition to amino acids and polypeptides, other types of biomaterials such as cellulose and vitamins are also widely used in PENGs. Bae and coworkers [109] extracted a chitin biofilm from black soldier fly (BSF) larvae. This biofilm has a nonporous and orderly repeating hexagonal structure. The TENG and PENG based on this chitin biofilm demonstrate excellent performance. The Ag/BSF/Ag PENG can generate a voltage of 18 V, a current of 2.2 μA, and an instantaneous power of 5 μW, showing that the chitin biofilm is an excellent material for developing sustainable energy harvesting devices and has broad application prospects in the field of wearable technology (Fig. 11B). Zhang et al. [110] fabricated biomass-based multilayer structured PENGs using a hydrogen bond replacement strategy. The matrix is a blend film of cellulose and PVDF, and the fillers are BCZT ceramic powders. The hydrogen bond network allows for uniform filler distribution. By changing the inner layer thickness, the output performance of PENG can be adjusted. For instance, the 1/3-0-1/3 configured device has a power density *P*_D_ of approximately 4.28 μW cm^−2^, and its *P*_D_/*F* (power density triggered by unit force) is the highest among reported cellulose-based PENGs. When applied to a pedometer, it shows high sensitivity and potential for working in humid environments (Fig. 11C).

Ji and colleagues [111] successfully prepared 20 high-quality vitamin crystals. The vitamin molecules in these crystals can self-assemble into 3 packing modes. DFT calculations showed that the piezoelectric strain constants of vitamin self-assemblies were from 3.8 to 42.8 pC N^−1^. The D-BIO (vitamin B7) self-assembly has a piezoelectric strain constant as high as 42.8 pC N^−1^ due to its low crystal symmetry and high polarization. The PENG fabricated based on D-BIO assemblies exhibits excellent performance. Under a mechanical force of 47 N, it generated an open-circuit voltage of approximately 0.8 V. After 5,400 pressing–releasing cycles, its performance remained stable for at least 3 months. The wearable sensor made from this PENG accurately detected the bending movements of human limbs. The insole based on PENG monitored different walking speeds and movement states in real time, and the generated electricity was sufficient to light up 12 LEDs, showing great potential in the field of self-powered devices (Fig. 11D). Typical biomaterials, performance, and applications of PENGs are shown in Table 1.

**Table 1.** Summary of biomaterial-based piezoelectric nanogenerators

Materials	Performance	Application	Ref.
γ-Glycine, PDMS	250 mV, 22.5 nA, 2.5 nW/cm^2^	Wound monitoring and healing	[163]
β-Glycine, chitosan	190 mV	Wearable biomedical diagnostics	[164]
Diphenylalanine peptide microrods	0.6 V, 7 nA, 0.1 nW	Flexible electronics, wearable devices, and soft robotics	[71]
Diphenylalanine peptide nanotubes	2.8 V, 37.4 nA, 8.2 nW	Energy harvesters	[70]
Black soldier Fly/PTFE (polytetrafluoroethylene)	18 V, 2.2 μA, 5 μW	Energy harvesting devices	[109]
Cellulose/PVDF	4.28 μW/cm^2^	Health monitoring and wearable electronics	[110]
Vitamin B_7_	1.19 nA, 0.061 μW/cm^2^	Wearable sensors, fitness tracking	[111]
Crab shell chitin nanofibers	22 V, 0.12 μA, 31.43 nW	Implantable medical goods	[165]
Four glutamates M13 bacteriophage nanopillars	232 mV, 11.1 nA, 0.99 nW	Self-powered implantable and wearable electronics	[166]
Fish scale	4 V, 1.5 μA, 1.14 μW/cm^2^	Self-powered implantable medical devices	[167]
Fish swim bladder	10 V, 51 nA, 4.15 μW/cm^2^	Self-powered biomedical sensors	[168]
Prawn shell chitin nanofibers	4.4 V, 3.7 nA, 0.76 μW/cm^2^	A human interactive self-powered wearable sensor	[169]
Onion skin	18 V, 166 nA, 1.7 μW/cm^2^	Pacemakers and healthcare units	[170]
Spider silk	21.3 V, 0.68 μA, 4.56 μW/cm^2^	Biomechanical energy harvesters	[76]
Collagen nanofibrils	45 V, 250 nA	Implantable devices, e-skin-based sensors and devices	[171]
Tomato peel	24.5 V, 2.5 μA, 19.5 μW	Portable and wearable applications	[172]
Pomelo fruit membrane	6.4 V, 7.44 μA, 12 μW/cm^2^	Health monitoring, the power source for the implantable devices	[173]
Wood sponge	0.69 V, 7.1 nA, 0.6 nW/cm^2^	A wearable movement monitoring system	[174]
Eggshell membrane	26.4 V, 1.45 μA, 11.90 μW/cm^2^	Self-powered implantable and wearable electronics	[175]
Chicken feather fibers	10 V, 21.6 mA, 6 μW/cm^2^	Wearable electronics, bio-implantable and healthcare monitoring devices	[176]
Ginkgo tree leaves	6.55 V, 125 mA, 1.29 μW	Self-sustainable wearable tactile sensors	[177]
BKH2 bacterial protein, PVDF	14 V, 168 nA, 64 μW/cm^2^	Clinical healthcare monitoring applications	[178]
Cotton, PVDF	65 V, 2.1 μA, 44.7 μW/cm^2^	Sustainable green energy harvesting	[179]
Chicken feather fibers, PVDF	28 V, 1.4 μW/cm^2^	Wearable bioelectronic patches and power sources	[180]
Snake ecdysis	15.5 V, 3.4 μA, 73 μW	Speakers, battery-free sensors, health monitoring devices	[181]
Sericin and fibroin	1.52 μW/cm^2^	Wearable sensors, energy harvesting	[182]

### Energy harvesting based on the triboelectric effect

#### Introduction of TENG

**Fig. 12.**
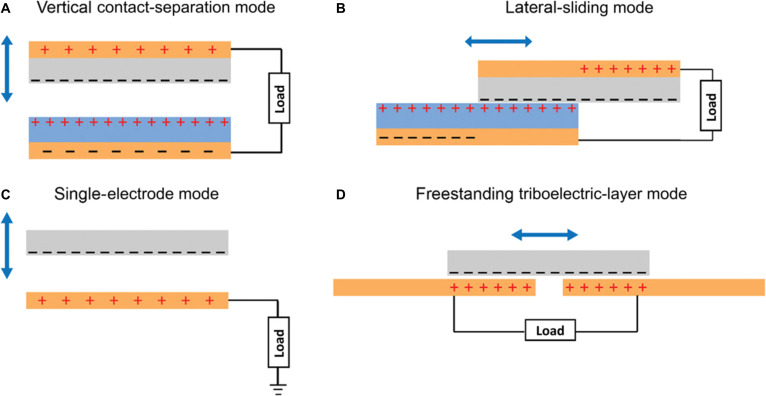
Four working modes of TENGs. (A) Vertical contact-separation mode. (B) Lateral-sliding mode. (C) Single-electrode mode. (D) Freestanding triboelectric-layer mode [112]. Adapted with permission of [112]. Copyright 2018 Wiley.

The TENG has been developed, which couples the contact electrification and the electrostatic induction between 2 different materials with different triboelectric polarities. This device exhibits several favorable properties, such as versatile structures and high voltage outputs. The triboelectric generators have 4 main working modes [112]: vertical contact-separation mode, lateral-sliding mode, single-electrode mode, and freestanding triboelectric-layer mode, as shown in Fig. 12. Since the triboelectric generator was first proposed, much research has mainly focused on improving the triboelectric charge density and energy conversion efficiency.

#### Biomaterial-based TENGs and applications

Polymers are usually used for the triboelectric negative layer of a TENG because of their easy incorporation of triboelectric negative atoms such as fluorine and nitrogen. However, polymer-based materials present problems of adverse impacts on the environment. Using biomaterials to construct TENGs is an excellent option, because biomaterials have good biocompatibility, degradability, and availability. Biomaterial-based TENGs may enable the development of wearable and implantable energy harvesters that can seamlessly integrate with biological systems. These materials are designed to be compatible with living organisms, minimizing any adverse effects on human health or the environment [113,114].

**Fig. 13.**
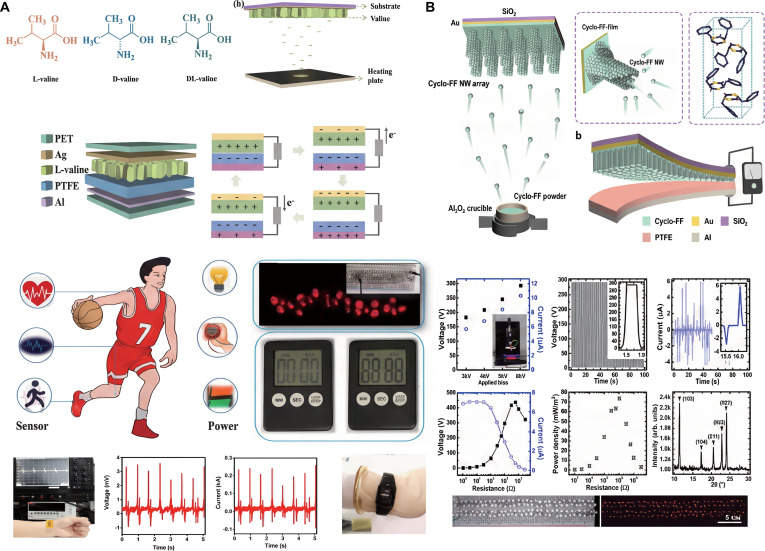
(A) Chiral valine self-assembly-based TENGs and their application [115]. Adapted with permission of [115]. Copyright 2023 Elsevier. (B) Cyclo-FF nanowire (NW) array-based TENGs and their performance [116]. Adapted with permission of [116]. Copyright 2019 Elsevier.

Our group successfully optimized the molecular spatial arrangement of valine using a 2-step approach involving chiral selection and electric field manipulation [115]. This technique effectively anchored the carboxyl end of the crystal to the substrate while exposing the amino group on the contact surface, leading to enhanced charge separation and markedly improved frictional performance, and the electric field can induce molecular orientation and effectively enhance frictional power generation performance. In addition to energy harvesting applications, the self-powered sensors developed in this study hold promise for detecting body movements such as walking, knee joint motion, and elbow motion. Furthermore, the TENG can be utilized to illuminate 24 red LEDs simply by tapping with the hand, and it can also power commercial timers and LCDs. This work underscores the potential of optimizing the crystal structure of biomaterials through careful material selection and electric field manipulation, thus paving the way for the design of high-performance bioelectronic devices with practical applications (Fig. 13A).

Biocompatible materials hold great promise for advancing biomedical applications. Among them, dipeptide nanostructures have attracted significant attention due to their biocompatibility, functional molecular recognition, and unique biological and electronic properties. However, the lack of stable arrays of peptide nanostructures that can be mass-produced and easily fabricated remains a major hurdle for practical implementation. To address this challenge, Heo and colleagues [116] developed a straightforward and scalable process for producing arrays of cyclohexyl phenylalanine (cyclo-FF) nanowires through thermal evaporation. They also successfully manufactured high-performance TENGs based on cyclo-FF. This innovative method enables precise control over the size of cyclo-FF nanowires and allows for the preparation of stable peptide nanostructures under varying environmental conditions and humidity levels. These cyclo-FF nanowires serve as the frictional material in biocompatible nanogenerators, showcasing impressive power output capabilities that hold tremendous potential for future applications (Fig. 13B).

Peptide-based core–shell structures hold great promise for various applications. In 2019, Wang and colleagues [117] synthesized peptide-Co_9_S_8_ nanobricks with a core–shell structure by covering self-assembled Co_9_S_8_ nanobelts with a thin shell layer using atomic layer deposition (ALD). The shell layer not only protects the peptide material from electrolyte attack but also provides additional capacitance for supercapacitors. Flexible asymmetric supercapacitors can be coupled with a TENG for a flexible self-powered TENG/SC (supercapacitor) system. The TENG/SC system, charged continuously by a TENG for 2.7 h, successfully powered a red LED for 21 min, demonstrating its excellent self-charging and power delivery performance. Protein is also an excellent friction layer material for a TENG. Genetically engineered spider silk protein was designed to improve triboelectric properties and mechanical strength [118]. Zheng and colleagues [119] prepared a high-performance TENG based on silk fibroin (SF) aerogel and polydimethylsiloxane (PDMS) sponge. Using silkworm cocoons as raw materials, SF aerogel with a porous structure was prepared by the directional freeze casting method. It was used as the positive friction layer, and the PDMS sponge was used as the negative friction layer. When the SF concentration was 3%, the TENG showed the best output performance, with a maximum open-circuit voltage of 365 V, a maximum short-circuit current of 11.8 μA, and a maximum power density of 7.52 W/m^2^. Compared with the TENG based on the SF film, the voltage is increased by 6.5 times and the current is increased by 4.5 times. This TENG can be used to monitor subtle human movements in real time, such as the movements of fingers, wrists, and knees, as well as activities like walking and swallowing, providing an effective solution for the development of wearable bioelectronic health monitoring devices.

**Fig. 14.**
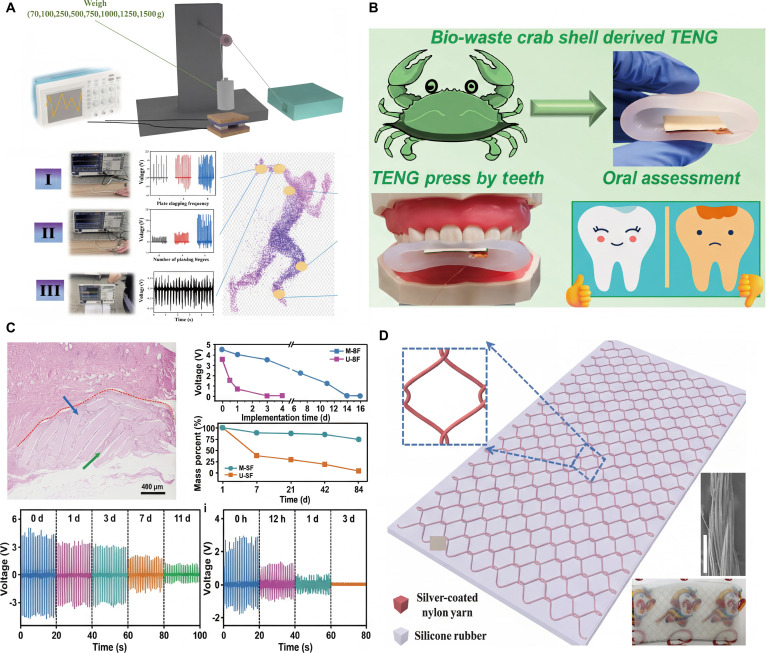
Other biomaterials used for TENGs. (A) Pd-absorbed PLA sheet, structural, and output performance of the as-fabricated TENG device [113]. Adapted with permission of [113]. Copyright 2023 American Chemical Society. (B) Systematic step-by-step synthesis of chitosan, gelatin, and cellulose and the demonstration of an oral healthcare sensor [120]. Adapted with permission of [120]. Copyright 2023 American Chemical Society. (C) Implantation schematic and electrical output of bioabsorbable natural materials-based TENGs [121]. Adapted with permission of [121]. Copyright 2018 Wiley. (D) Schematic illustration of the skin-inspired triboelectric nanogenerator (SI-TENG) with “chain-link” fence-shaped structure and rhombic unit design, and the applications of the SI-TENG [122]. Adapted with permission of [122]. Copyright 2018 Wiley.

Chitosan, gelatin, cellulose, and nylon are also used to make TENGs. Gui and colleagues [113] developed a bio-based TENG with PLA as the base material. Micropores and hydrophilic groups such as hydroxyl groups and carboxyl groups were introduced onto the surface of PLA through alkali treatment, and then a nickel layer was deposited on its surface as the electrode material by electroless nickel plating. Using PDMS and PVA as the friction layers, a TENG working in a vertical contact-separation mode was constructed. Under a 6% strain (corresponding to a stress of 32.4 kPa), the device produced a maximum output voltage of approximately 5.1 V. It was found that within the stress range of 0.14 to 32.4 kPa and the strain range of 1.3% to 6%, the output voltage has a linear regression relationship with the strain of the dielectric material, and the electrostatic charge density formed on the TENG surface is 4.1 × 10^6^ C/m^2^ (Fig. 14A).

Kim and colleagues [120] prepared a TENG for oral health monitoring using biological waste. Chitosan, gelatin, and cellulose were extracted from crab shells, fish scales, and wood waste and compounded with biodegradable PVA. With rice paper (RP) as the substrate and edible silver leaf as the electrode, a single-electrode mode TENG was constructed. Among them, the PVA/chitosan 10 wt % composite-based TENG (PC10) performed the best, with an output voltage of approximately 20 V, a current of 200 nA, and a charge of 12 nC. This TENG can collect biomechanical energy to power LEDs. A biocompatible bite sensor based on a TENG was also fabricated to measure the bite force of a denture model, thereby evaluating oral health conditions and providing a promising direction for the development of disposable oral medical devices (Fig. 14B). In another work, Li and colleagues [121] utilized the highly biodegradable natural polymer cellulose, chitin, SF, RP, and egg white (EW) to prepare bioresorbable natural materials-based TENGs (BN-TENGs) (Fig. 12). A wide range of electrical output was achieved by BN-TENGs, with *V*_oc_ ranging from 8 to 55 V and *I*_sc_ ranging from 0.08 to 0.6 μA, respectively. The BN-TENG served as a voltage source to power an extracorporeal electrical stimulation system and successfully regulated the function of isolated clusters of dysfunctional cardiac muscle cells. Given its impressive in vitro and in vivo electrical output, excellent biocompatibility, biodegradability, and tunable bioabsorbability, it holds tremendous potential as a transient power source for electronic and bioabsorbable implantable medical devices. Nylon, which has excellent mechanical robustness and softness, has a strong electron-donating ability compared with metal, and is often used as a positive electrode material in the TENG (Fig. 14C) [121]. Wang and colleagues [122] developed a stretchable and washable skin-inspired TENG made by embedding a planar conductive yarn network composed of 3-ply-twisted silver-coated nylon yarn. This device combines the functions of biomechanical energy harvesting and multifunctional pressure sensing, and has good stretchability, sensitivity, detection accuracy, response speed, and mechanical stability. Its maximum average power density reaches 230 mW/m^2^, which can light up 170 LEDs, charge capacitors, and drive small electronic products. As a self-powered multifunctional sensor, it can monitor human physiological signals and is also used to fabricate intelligent prosthetic hands, self-powered pedometers/speedometers, flexible digital keyboards, and pressure sensing arrays with 8 × 8 pixels, showing broad application prospects in fields such as wearable power supply technology, physiological monitoring, intelligent prosthetics, and human–machine interfaces (Fig. 14D).

PENGs have a wide range of applications and offer several advantages in various fields (Table 2). They can harvest energy from a great range of energy sources, such as human motion, machine vibrations, wind blow, and water flow. TENGs provide sustainable energy supply for small electronic devices, sensors, and wearable technologies.

**Table 2.** Summary of biomaterial-based triboelectric nanogenerators

Materials	Performance	Application	Ref.
Poly(viny alcohol) (PVA)/chitosan	20 V, 200 nA, and 12 nC	Oral health monitoring, bite force sensing, disposable medical devices	[120]
Silk fibroin (SF)/PDMS	365 V, 11.8 μA, 7.52 W/m^2^	Wearable electronics	[119]
Chicken skin	123 V and 20 μA from a 9 cm^2^ device	Capacitors, power supplies, electronic calculators, LED	[183]
WPI (whey protein isolate)	4 V, 2.8 W/m^2^	Wearable sensor	[184]
Silk protein	Resolution of 0.1 Hz and sensitivity of 167 mV/dB	Authentication, information security, high-fidelity platforms, machine control, and medical rehabilitation	[185]
Silk and PET, electrospun nylon 66 nanofibers, PVDF-coated PET fabric	100 V, 24.5 mA/m^2^	Wearable electronics	[186]
SFF (silk fibroin film)	13 V, 0.4 μA, 0.8 W/m^2^	Wearable electronics	[187]
bR protein, CNTs	11 nA/cm^2^, 0.43 mW/cm^2^	Light conversion systems	[188]
Ostrich EM	300 V, 0.18 mW	Body motion detection	[189]
Sodium alginate (SA)/PVA	203.4 V, 17.6 μA, 0.98 mW/cm^2^	Wearable electronics	[190]
PEO-silk nanofibers	2.1 kV, 6.5 μA, 196 mW/cm^2^	Wearable electronics	[191]
FG (fish gelatin) PTFE/PDMS	130 V, 0.35 μA, 45.8 μW/cm^2^	Wearable electronics	[192]

### Energy harvesting based on the hydrovoltaic effect

#### Introduction and mechanism of hydrovoltaic

**Fig. 15.**
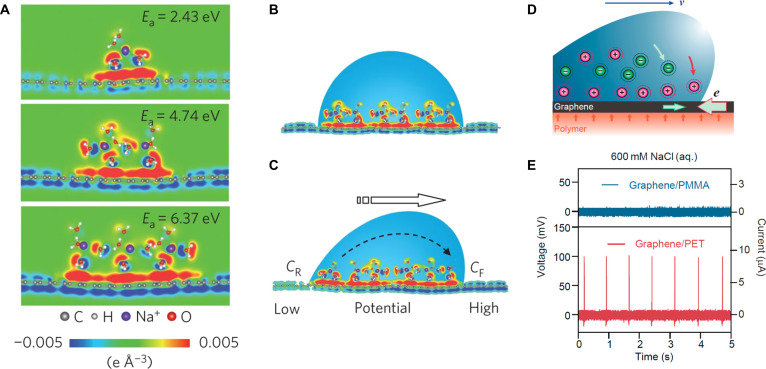
Mechanism for the drawing potential. (A) DFT results of the differential charge distribution near graphene caused by adsorbing 1 to 3 rows of hydrated sodium cations. The corresponding adsorption energy (*E*_a_) is provided. (B) Schematic illustration of the pseudocapacitance formed by a static droplet on graphene. (C) Schematic illustration of the potential difference induced by a moving droplet [123]. Adapted with permission of [123]. Copyright 2014 Springer. (D) Schematic motion of ions in solution and electrons in graphene toward the front edge of the droplet in response to an ionic droplet moving forward with velocity *v*. (E) Voltage and current outputs of a ferroelectric polymer film [124]. Adapted with permission of [124]. Copyright 2018 American Chemical Society.

In 2014, Guo and colleagues [123] first discovered that aqueous droplets containing Na^+^ ions can generate electrical signals by moving on the surface of single-layer graphene. The phenomenon of stable output of electrical energy through interaction with water in carbon nanomaterials is called the “hydrovoltaic effect” (Fig. 15). Ions are drawn to graphene/polymer interface by the polymer’s polar surface dipole layer. As droplets move, ions charge at the advancing front and discharge at the receding edge, inducing current in graphene via carrier movement [124]. Therefore, using ferroelectric thin films with strong surface dipoles can improve the electricity generation performance. Rainwater in nature contains abundant ions, and the sliding of raindrops on the surface of functional materials is expected to electricity.

#### Biomaterials for hydrovoltaic technology and applications

**Fig. 16.**
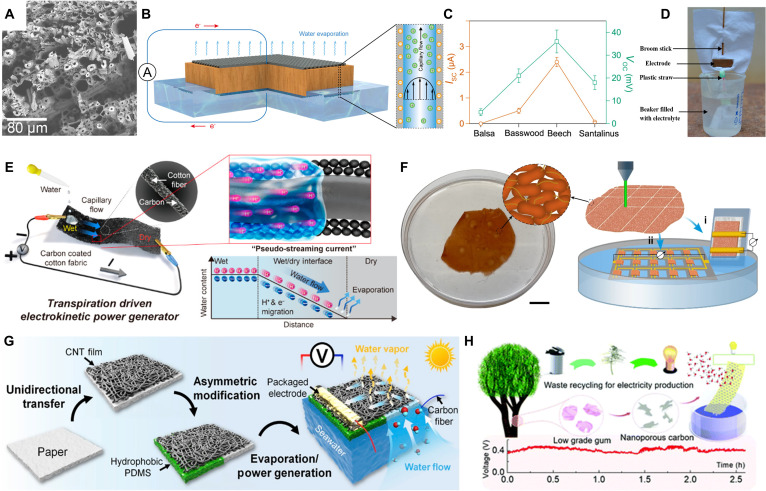
(A) SEM image of the top view of beech wood. (B) Schematic illustration of the electricity generation mechanism driven by water evaporation from vertically aligned microchannels in natural wood. Two electrodes are connected to the wood’s top and bottom ends specifically for measuring the electrical signal output. (C) Short-circuit current and open-circuit voltage of beech wood versus CA modification reaction time [125]. Adapted with permission of [125]. Copyright 2020 American Chemical Society. (D) Complete fabric channel (FC)-based generator, demonstrating its various segments [126]. Adapted with permission of [126]. Copyright 2019 American Chemical Society. (E) Schematic illustration of the operation method of transpiration-driven electrokinetic power generator (TEPG) composed of carbon black-coated cotton fabric, as well as the pseudostreaming behavior that occurred at the wet/dry interface and the mechanism of current generation [127]. Adapted with permission of [127]. Copyright 2022 Springer. (F) Schematic illustration of the operation of carbon black-coated cotton fabric [129]. Adapted with permission of [129]. Copyright 2022 Springer (G) Fabrication of CNTs/PDMS/paper hybrid as an all-in-one evaporator that is capable of simultaneous water evaporation and electricity generation [130]. Adapted with permission of [130]. Copyright 2020 Elsevier. (H) Fabrication process of a hydrovoltaic device based on gum-derived carbon to generate power [131]. Adapted with permission of [131]. Copyright 2023 RSC.

Biomaterials are mainly composed of carbon, hydrogen, and oxygen. Without undergoing any chemical changes, they are readily broken down by organic microbes into smaller molecules like carbon dioxide and water, and the byproducts they produce can reenter the natural cycle. Hence, products derived from natural wood, cotton, and other biomass have exceptional renewability and biodegradability. Natural wood is rich of hydroxyl groups, which primarily made up of cellulose, lignin, and hemicellulose. Zhou et al. [125] employed citric acid (CA)-modified beech wood to harvest electricity from water evapotranspiration based on the capillarity phenomenon. Wood’s continuous microchannels had an average diameter of roughly 10 μm (Fig. 16A). The energy harvester featured a classic sandwich configuration. In the vertical direction relative to the wood, 2 polyethylene terephthalate (PET) meshes with conductive carbon paste coatings served as external electrodes and 2 inert electrodes were inserted into the reservoirs located at the capillary’s 2 ends (Fig. 16B). As water passed through the wood microchannels, the hydroxyl groups on the channel walls were hydrolyzed to render the channels negatively charged which, generated a 300-mV open-circuit voltage, a 10-μA short-circuit current, and a power density of 0.045 μW/cm^2^ (Fig. 16C). The same phenomenon was also observed in other wood species, which made them have tremendous potential in nanogenerator application. Das et al. [126] developed an innovative energy harvesting system utilizing a commercial wearable fabric. This system mimics the natural process of plants by channeling water through the fabric’s nanochannels, thereby generating electricity through the combined effects of transpiration and capillary action (Fig. 16D). Yun et al. [127] developed a type of wetted cotton fabric reinforced with carbon black and achieved a potential difference of 0.53 V between the asymmetric wet and dry sides of the cotton fabric by using the water’s capillary movement (Fig. 16E).

Chen et al. [128] reported macroscale evaporation-driven engines that can power common tasks like locomotion and electricity generation. With these engines, they exhibited an electricity generator that rests on water while harvesting its evaporation to power a light source, and a miniature car (weighing 0.1 kg) that moves forward as the water in the car evaporates. Liu et al. [129] fabricated a device with microbial biofilms made from sustainable feedstocks to produce electricity from water evaporation (Fig. 16F). Xiao et al. [130] developed a bilayer solar evaporator that effectively implemented the directed water flow (Fig. 16G). To generate centimeter-sized water flow channels, large-area CNT films were applied to the cellulose paper’s surface, and hydrophobic PDMS was enclosed at a single spot on one side of the paper, leading to the formation of an asymmetric alteration. It generated a maximum output power of 2.1 mW as water evaporated under solar radiation. Venkateshaiah et al. [131] discovered that nonfood-grade tree gum could be converted into high-quality carbon materials with a high specific surface area and a large number of micro/mesopores through carbonization and exfoliation. The discoveries of biomolecular self-assembled materials advances the development of environmentally friendly materials for the fabrication of hydrovoltaic devices, advancing the energy conversion technology based on the hydrovoltaic effect (Table 3).

**Table 3.** Summary of biomaterial-based hydrovoltaic devices

Materials	Performance	Application	Ref.
Citric acid-modified wood	300 mV, 10 mA, 0.045 μW/cm^2^	Nanogenerator	[125]
Wet fabric pieces	700 mV	Enlight white LED	[126]
Bacterial spores	60 μW	Evaporation-driven car and powering of LEDs	[128]
Microorganisms	1 μW/cm^2^	Electricity generation	[129]
Carbon black-coated cotton fabric	0.53 V, 3.91 mA	Turning on a LED	[127]
Bilayer (CNTs) film/cellulose paper	2.1 μW	Electricity generation	[130]
Nanoporous carbon	0.4 V, 3 mA	Turning on a LED	[131]
Protein nanowires	0.5 V, 17 μA/cm^2^	Power source	[193]
Waste corn stalk	0.6 V, 0.5 μA/cm^2^	Power source for small electronics	[194]
Electrospun cellulose acetate	0.3 V, 80 nA/cm^2^	Power source for LCD	[195]
Gelatin	0.71 V, 7.77 μA/cm^2^	Smart mask	[196]
Nanofibrils	0.1 V, 30 nA/cm^2^	Body motion sensor	[197]
Print paper	0.25 V, 30 nA/cm^2^	Green power supply	[198]

### Other energy harvesting

#### Biosolar cells

**Fig. 17.**
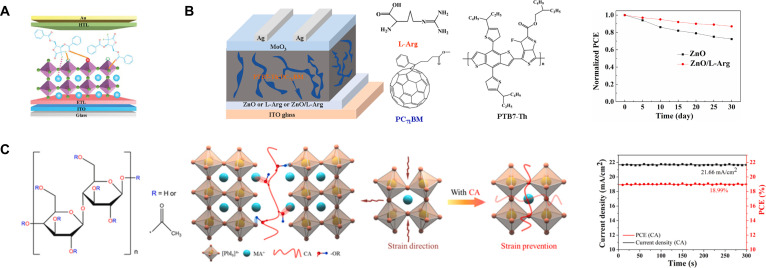
(A) Schematic diagram of the device structure with the top surface of perovskite modified by DDPUD [134]. Adapted with permission of [134]. Copyright 2024 Wiley. (B) Schematic diagram of OSCs and the chemical structures of l-arginine (l-Arg), PTB7-Th, and PC71BM. Curves show the variation of PCE of OSCs with storage time when ZnO and ZnO/l-Arg are used as ETLs, respectively [135]. Adapted with permission of [135]. Copyright 2020 Elsevier. (C) Chemical structure of CA and schematic diagram showing the expansion/shrinkage of perovskite crystal lattices without and with CA during the annealing process. Maximal steady-state photocurrent and stabilized PCE of optimal CA-treated PSCs [136]. Adapted with permission of [136]. Copyright 2021 Elsevier.

Biomaterials, featuring nontoxicity, sustainability, and cost-effectiveness, have emerged as promising candidates for advancing the performance and stability of optoelectronic devices, particularly solar cells [e.g., perovskite solar cells (PSCs), organic solar cells (OSCs), and dye-sensitized solar cells (DSCs)] and perovskite LEDs (PeLEDs) [132,133]. In PSCs, Chen and colleagues [134] developed a polydentate chelating biomaterial, 2-deoxy-2,2-difluoro-d-erythro-pentafuranous-1-ulose-3,5-dibenzoate (DDPUD), to stabilize the top interface of n-i-p PSCs (Fig. 17A). DDPUD chemically anchors to uncoordinated Pb^2+^, halide vacancies, and I-Pb antisite defects, reducing trap density (from 2.01 × 10^16^ to 1.49 × 10^16^ cm^−3^) and releasing interfacial tensile strain. PSCs fabricated in ambient air with DDPUD achieved a power conversion efficiency (PCE) of 24.47%, among the highest for air-processed device.

Natural amino acids and their derivatives have also shown efficacy in OSCs and PSCs. Li et al. [135] employed l-arginine (l-Arg) as an electron transport layer (ETL) in inverted OSCs based on PTB7-Th:PC₇₁BM. l-Arg reduces the work function of indium tin oxide (ITO) via interfacial dipoles, improving energy-level alignment with PC₇₁BM. When combined with ZnO as a double ETL (ITO/ZnO/l-Arg), the device PCE reached 9.31% (versus 8.09% for pure ZnO), with reduced series resistance (3.71 Ω·cm^2^) and enhanced stability (Fig. 17B). For PSCs, cellulose derivatives [e.g., cellulose acetate (CA) and cellulose acetate butyrate (CAB)] act as dual-functional additives. Xiong and colleagues [136] reported that CA forms hydrogen bonds with MA^+^ and I^−^ in MAPbI, promoting crystal growth and achieving a PCE of 19.53% with 15-d stability (Fig. 17C). CAB, with lower moisture absorption, passivates Pb^2+^ via ester groups and I^−^ via hydroxyl groups, boosting PCE to 21.5% and maintaining 90% initial efficiency after 3,300 h at 35% relative humidity (RH).

Biomaterials also find applications in other device components, such as substrates and electrodes. Transparent wood (TW), prepared by delignification and polymer infiltration, serves as a PSC substrate with 86% transmittance at 550 nm, achieving a PCE of 16.8% [137]. Biochar, derived from brewery residues or soybean dregs, replaces noble metal (Pt, Au) electrodes in DSCs and PSCs: Miettunen and colleagues [138] used brewery residue-derived biochar as a DSC counter-electrode, showing slower electrolyte degradation than Pt; Ma and colleagues [139] reported that soybean dreg-derived N/O-co-doped biochar enhances PSC stability, retaining 87% PCE after 2,000 h. Overall, biomaterials offer a sustainable pathway to high-performance, eco-friendly optoelectronic devices, paving the way for their commercialization.

#### Biofuel cells

BFCs, as promising energy harvesting devices that convert chemical energy from biological substrates into electricity, have garnered intensive research interest for powering implantable electronics, environmental remediation, and sustainable energy supply [140,141]. For implantable BFCs, especially those targeting soft tissues like the brain, mechanical flexibility and anti-biofouling properties are paramount. Peng and colleagues [142] developed a flexible fiber BFC using CNT fiber as the electrode substrate, which exhibits excellent mechanical match with brain tissues (bending stiffness comparable to biological tissues) and structural integrity even after knotting. To mitigate biofouling, a hydrophilic zwitterionic polydopamine-2-methacryloyloxyethyl phosphorylcholine (PDA-MPC) layer was in situ polymerized on the fiber surface, effectively resisting nonspecific protein adsorption. In vivo tests demonstrated that this BFC stably generated power in the mouse brain for over a month, with a maximum power density of ~4.4 μW cm^−2^ in vivo and 32.6 μW cm^−2^ in vitro, while eliciting negligible immune response (no significant astrocyte/microglia accumulation or neuron depletion).

Microbial-based BFCs have benefited from biomaterial modification to enhance electron transfer. Kavaliauskaitė et al. [143] proposed a cost-effective modification of *Saccharomyces cerevisiae* with Prussian Blue (PB) using FeCl₃ and K₃[Fe(CN)₆] salts. Optical microscopy and gravimetric assays confirmed PB formation in the yeast cell wall/periplasm without compromising viability. Cyclic voltammetry (CV) showed that PB-modified yeast exhibited a higher anodic peak current (617 μA cm^−2^) than unmodified yeast (547 μA cm^−2^), as PB acted as an electron transfer mediator between cell membrane oxidoreductases and [Fe(CN)₆]^3−^. The resulting BFC achieved a maximum power density of 58.3 mW m^−2^, 24% higher than that of unmodified yeast.

Other biomaterials also provide multiple pathways for improving the performance of BFCs, such as microalgae and lignin. Microalgae, as photosynthetic biomaterials, offer versatile pathways for BFC fuel production and integration. Luque and colleagues [144] reviewed thermochemical [pyrolysis, gasification, hydrothermal liquefaction (HTL)] and biochemical (fermentation) conversion of microalgae (e.g., *Chlorella vulgari*) into biofuels. Lignocellulosic biomass, as a renewable feedstock, was integrated with BFCs via thermochemical conversion. Park and colleagues [145] reported gasification-based integrated systems [e.g., biomass gasification + solar thermal energy and solid oxide fuel cell (SOFC)]. In conclusion, biomaterials have markedly advanced BFC performance in flexibility, anti-biofouling, electron transfer, and fuel versatility. However, challenges remain, including large-scale fabrication, long-term in vivo stability, and cost reduction.

#### Biomaterial-based TEGs

TEGs have emerged as a promising technology for sustainable energy harvesting, and the integration of biomaterials has significantly advanced their flexibility, biocompatibility, and environmental sustainability. Biomaterials, derived from natural sources or biodegradable components, address key limitations of traditional inorganic thermoelectric (TE) materials—such as rigidity, toxicity, and high production costs—while enabling novel applications in wearable devices, off-grid power supply, and biomedical systems [146,147].

Cellulose, a renewable and abundant biomass, has become a cornerstone in biomaterial-based TEGs due to its hierarchical structure, tunable porosity, and excellent mechanical adaptability. A hierarchically porous cellulose membrane (HPCM) fabricated via molecular self-assembly engineering exhibits ~95% porosity, >94% solar reflectance, and >0.9 mid-infrared emissivity [148]. When integrated into a cellulose-based thermoelectric (CTE) module, the HPCM acts as a passive radiative cooler, inducing an average temperature gradient of 14.5 °C during daytime natural convection—17-fold higher than pristine devices. Another cellulose-based innovation is a self-powered TE gel composed of microcrystalline cellulose (MCC) and poly(deep eutectic solvent) (PDES), synthesized via a one-pot method [149]. This gel combines the sustainability of MCC with the ionic conductivity of PDES, achieving a Seebeck coefficient of 25.86 mV K^−1^, low thermal conductivity, and a ZT (thermoelectric figure of merit, a dimensionless parameter evaluating the thermoelectric energy conversion efficiency) value of 0.27 at room temperature.

Polymer–biomass composites integrate the flexibility of polymers with the sustainability of biomass, enabling TEGs for all-weather energy harvesting. A bioinspired micro/nanostructured polyethylene/poly(ethylene oxide)/graphene (MN-PPG) film, fabricated via industrialized microextrusion compression molding (μ-ECM), exemplifies this approach [150]. The film leverages polyethylene (PE) for mechanical stability, poly(ethylene oxide) (PEO) as a phase-change material for energy storage, and graphene for photothermal conversion. Its micro/nanostructured surface confers superhydrophobicity (contact angle ~156°) and antireflectivity, boosting light absorption. Under 3-sun irradiation, the integrated TEG generates an open-circuit voltage of 315.4 mV and a power density of 2.5 W m^−2^; at night, it recycles afterheat from LED chips, realizing round-the-clock energy circulation.

Biomaterials have revolutionized TEG design by enabling sustainability, flexibility, and biocompatibility. From cellulose-based porous membranes for off-grid power to polymer–biomass composites for all-weather harvesting and biomedical-grade films for theranostics, these materials address critical limitations of traditional TEGs. As research advances in performance optimization and scalability, biomaterial-based TEGs will play a pivotal role in sustainable energy systems and personalized healthcare, contributing to a low-carbon and resource-efficient future.

## Conclusion and Perspectives

### Conclusion

**Fig. 18.**
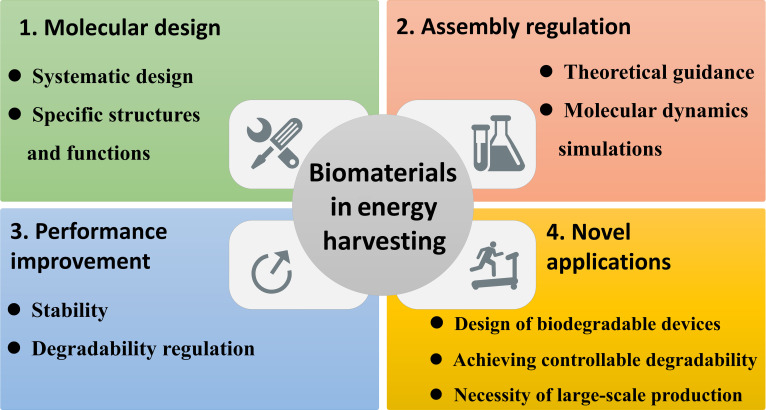
Perspectives of the biomaterial in energy harvesting.

Biomolecular self-assembly presents an effective strategy to produce functional bionanomaterials. Because of the easy synthesis process, various shapes, tunable structures, and versatile properties, self-assembled biomaterials are excellent candidates for the study of intermolecular interactions. In terms of self-assembly mechanisms, this review focuses on elaborating the core methods and key forces that drive the self-assembly of biomolecules. These forces include chemical bond interactions such as hydrogen bonds and covalent bonds, as well as noncovalent interactions like π–π stacking, electrostatic attraction, and hydrophobic effects. In addition, this review conducts a detailed analysis of the structural characteristics and diverse properties of different types of self-assembled biomolecular materials. In terms of mechanical properties, such materials exhibit strength and toughness suitable for nanoscale applications; in terms of electrical properties, their piezoelectric, ferroelectric, and semiconductor characteristics endow them with the potential for energy conversion; meanwhile, the core advantage of biocompatibility ensures the safe application of these materials in fields such as biomedicine. The combination of these properties makes self-assembled biomolecular materials an ideal choice for interdisciplinary applications. In practical applications, the review focuses on the key role of self-assembled biomolecular materials in the field of energy harvesting, particularly their applications in PENGs, TENGs, water-enabled electricity generation, and other types of energy harvesting devices. By utilizing the piezoelectric and triboelectric properties of the materials, mechanical energy in the environment (such as vibration and friction) can be efficiently converted into electrical energy, providing sustainable energy for microelectronic devices and wearable devices. In summary, this review not only clarifies the versatility of self-assembled biomolecules but also reveals their great potential in the innovative design of energy harvesting systems and bio-based microelectronic devices. This research outcome provides new ideas for the innovative design of biomaterials, is expected to promote technological breakthroughs in related fields, facilitates the transformation of biomolecular self-assembly technology from basic research to practical applications, and paves the way for the development of sustainable energy and bioelectronic devices (Fig. 18).

### Perspectives

1. Molecular design and assembly regulation: The limited knowledge of the self-assembly potential of different kinds of biomolecules and the lack of fundamental understanding of the self-assembly process markedly restricted the development of new biomaterials. Experimental and theoretical research efforts are needed to examine the self-assembly process of amino acids, peptides, protein, metabolites, and other biomolecules, establishing the foundation for the design and fabrication of functional self-assembled biomaterials.
2. Performance and long-term stability: In addition to further improving the mechanical, piezoelectric, and other physical properties, addressing the fundamental challenges of energy conversion efficiency, power output density, and long-term stability is paramount for translating biomolecular self-assemblies into practical devices. Self-assembled biomaterial-based devices exhibit significantly lower power densities than traditional technologies. To narrow this gap, synergistically modifying with piezoelectric/conductive fillers, optimizing device structures to improve interfacial charge transfer, and constructing piezoelectric–triboelectric hybrid systems to compensate for single-mechanism limitations are effective strategies [151–153]. While biomaterial-based devices lag behind synthetic inorganic materials in conversion efficiency, their core value lies in biocompatibility-dependent and context-appropriate energy output scenarios. An integrated approach combining performance enhancement, stability assurance, and lifecycle regulation will facilitate their transition from laboratory research to practical use. Additionally, their operational stability in complex humid environments remains a key bottleneck. To overcome this, material engineering strategies like chemical cross-linking and the development of robust composite materials are useful strategies [154,155]. Furthermore, biomaterials are prone to degradation under harsh conditions, leading to mechanical strength loss after wearable use and power drop after water immersion. To address this problem, physical surface coating or waterproof encapsulation layers can shield these delicate structures from degradation [156].
3. Practical applications: Although many applications have been proposed and demonstrated for biomolecular selfassemblies, many challenges need to be overcame before they can be put into practical usage. The first challenge will be the massive production of high-quality materials. Most synthesis methods used in laboratory may not be adopted for industrial production. In addition, the impact of selfassembled micro- and nanostructures for the living system are largely unknown. To address this, researchers can explore low-cost biomass precursors and green solvents to reduce costs, and develop continuous-flow microfluidic self-assembly technologies [157,158] to precisely regulate intermolecular interactions for large-volume uniform production. Moving from laboratory synthesis to industrial production requires improving the controllability of self-assembly and batch-to-batch consistency. This can be achieved by optimizing reaction parameters through machine learning-driven design and implementing automated quality control to validate product uniformity [159,160]. Standardization of performance evaluation for bio-based energy harvesters is another key hurdle, as a lack of unified metrics hinders fair comparison and optimization.To address this issue, a consensus-based framework can be developed through interdisciplinary collaboration to define the core metrics for systematic evaluation. Furthermore, integration with existing microelectronic systems needs dedicated research for seamless functionality. Creating compatible interface architectures can bridge the material and functional gaps. Beyond manufacturing and standardization, biological implications require thorough investigation. The establishment of a multidimensional biocompatibility assessment platform allows for the characterization of immune responses and informs risk-mitigating structural modification.

## References

[1] Irimia-Vladu M. “Green” electronics: Biodegradable and biocompatible materials and devices for sustainable future. Chem Soc Rev. 2014;43(2):588–610. 24121237 10.1039/c3cs60235d

[2] Chen C, Guo H, Chen L, Wang YC, Pu X, Yu W, Wang F, Du Z, Wang ZL. Direct current fabric triboelectric nanogenerator for biomotion energy harvesting. ACS Nano. 2020;14(4):4585–4594. 32181639 10.1021/acsnano.0c00138

[3] Yin J, Jia P, Ren Z, Zhang Q, Lu W, Yao Q, Deng M, Zhou X, Gao Y, Liu N. Recent advances in self-powered sensors based on ionic hydrogels. Research. 2025;8:0571. 39810855 10.34133/research.0571 PMC11729273

[4] Jiang W, Liu C, Liu W, Zheng L. Advancements in intelligent sensing technologies for food safety detection. Research. 2025;8:0713. 40458611 10.34133/research.0713 PMC12128931

[5] Sakuragi T, Nagata S. Regulation of phospholipid distribution in the lipid bilayer by flippases and scramblases. Nat Rev Mol Cell Biol. 2023;24:576–596. 37106071 10.1038/s41580-023-00604-z PMC10134735

[6] Zhu J, Avakyan N, Kakkis A, Hoffnagle AM, Han K, Li YY, Zhang ZY, Choi TS, Na Y, Yu CJ, et al. Protein assembly by design. Chem Rev. 2021;121(22):13701–13796. 34405992 10.1021/acs.chemrev.1c00308 PMC9148388

[7] Yang D, Zhou C, Gao F, Wang P, Ke Y. DNA-guided assembly of molecules, materials, and cells. Adv Intell Syst. 2020;2(1):1900101.

[8] Ford JE, Stansfeld PJ, Vakonakis I. Coupling form and function: How the oligomerisation symmetry of the SAS-6 protein contributes to the architecture of centriole organelles. Symmetry. 2017;9(5):74.

[9] Xie W, Wei X, Kang H, Jiang H, Chu Z, Lin Y, Hou Y, Wei Q. Static and dynamic: Evolving biomaterial mechanical properties to control cellular mechanotransduction. Adv Sci. 2023;10(9):2204594. 10.1002/advs.202204594 PMC10037983 36658771

[10] Kim D, Han SA, Kim JH, Lee J-H, Kim S-W, Lee S-W. Biomolecular piezoelectric materials: From amino acids to living tissues. Adv Mater. 2020;32(14):1906989. 10.1002/adma.201906989 32103565

[11] Moura BS, Monteiro MV, Ferreira LP, Lavrador P, Gaspar VM, Mano JF. Advancing tissue decellularized hydrogels for engineering human organoids. Adv Funct Mater. 2022;32(29):2202825.

[12] Wang L, Gong C, Yuan X, Wei G. Controlling the self-assembly of biomolecules into functional nanomaterials through internal interactions and external stimulations: A review. Nano. 2019;9(2):285–311. 10.3390/nano9020285 PMC6410314 30781679

[13] Wang J, Liu K, Xing R, Yan X. Peptide self-assembly: Thermodynamics and kinetics. Chem Soc Rev. 2016;45(20):5589–5604. 27487936 10.1039/c6cs00176a

[14] Zou P, Chen W-T, Sun T, Gao Y, Li L-L, Wang H. Recent advances: Peptides and self-assembled peptide-nanosystems for antimicrobial therapy and diagnosis. Biomater Sci. 2020;8(18):4975–4996. 32931527 10.1039/d0bm00789g

[15] Bauri K, Nandi M, De P. Amino acid-derived stimuli-responsive polymers and their applications. Polym Chem. 2018;9(11):1257–1287.

[16] Raghupathi KR, Guo J, Munkhbat O, Rangadurai P, Thayumanavan S. Supramolecular disassembly of facially amphiphilic dendrimer assemblies in response to physical, chemical, and biological stimuli. Acc Chem Res. 2014;47(7):2200–2211. 24937682 10.1021/ar500143u PMC4100797

[17] Kumar M, Brocorens P, Tonnelé C, Beljonne D, Surin M, George SJ. A dynamic supramolecular polymer with stimuli-responsive handedness for in situ probing of enzymatic ATP hydrolysis. Nat Commun. 2014;5(1):5793. 25511998 10.1038/ncomms6793

[18] Habibi N, Kamaly N, Memic A, Shafiee H. Self-assembled peptide-based nanostructures: Smart nanomaterials toward targeted drug delivery. Nano Today. 2016;11(1):41–60. 27103939 10.1016/j.nantod.2016.02.004 PMC4834907

[19] Gatto E, Toniolo C, Venanzi M. Peptide self-assembled nanostructures: From models to therapeutic peptides. Nano. 2022;12(3):466. 10.3390/nano12030466 PMC8838750 35159810

[20] Clark TD, Buriak JM, Kobayashi K, Isler MP, McRee DE, Ghadiri MR. Cylindrical β-sheet peptide assemblies. J Am Chem Soc. 1998;120(35):8949–8962.

[21] Abbas M, Zou Q, Li S, Yan X. Self-assembled peptide- and protein-based nanomaterials for antitumor photodynamic and photothermal therapy. Adv Mater. 2017;29(12):1605021. 10.1002/adma.201605021 28060418

[22] Araya-Chavarría K, Rojas R, Ramírez-Amador K, Sulbarán-Rangel B, Rojas O, Esquivel-Alfaro M. Cellulose nanofibers as functional biomaterial from pineapple stubbles via TEMPO oxidation and mechanical process. Waste Biomass Valori. 2022;13(3):1749–1758.

[23] Chen C, Kuang Y, Zhu S, Burgert I, Keplinger T, Gong A, Li T, Berglund L, Eichhorn SJ, Hu L. Structure–property–function relationships of natural and engineered wood. Nat Rev Mater. 2020;5(9):642–666.

[24] Dong XY, Liu Q, Liu S, Wu RH, Ma LY. Silk fibroin based conductive film for multifunctional sensing and energy harvesting. Adv Fiber Mater. 2022;4(4):885–893.

[25] Mahadevi AS, Sastry GN. Cooperativity in noncovalent interactions. Chem Rev. 2016;116(5):2775–2825. 26840650 10.1021/cr500344e

[26] Rahsepar FR, Moghimi N, Leung KT. Surface-mediated hydrogen bonding of proteinogenic alpha-amino acids on silicon. Acc Chem Res. 2016;49(5):942–951. 27014956 10.1021/acs.accounts.5b00534

[27] Yuan H, Xue B, Yang D, Rencus-Lazar S, Cao Y, Gazit E, Tan D, Yang R. Rational design of biological crystals with enhanced physical properties by hydrogen bonding interactions. Research. 2023;6:0046. 36930775 10.34133/research.0046 PMC10013789

[28] Yuan H, Chen Y, Lin R, Tan D, Zhang J, Wang Y, Gazit E, Ji W, Yang R. Modified Stranski–Krastanov growth of amino acid arrays toward piezoelectric energy harvesting. ACS Appl Mater Interfaces. 2022;14(41):46304–46312. 36196653 10.1021/acsami.2c13399

[29] Zhang H, Wu Z, Zhou J, Wang Z, Yang C, Wang P, Fareed MS, He Y, Su J, Cha R, et al. The antimicrobial, hemostatic, and anti-adhesion effects of a peptide hydrogel constructed by the all-d-enantiomer of antimicrobial peptide Jelleine-1. Adv Healthc Mater. 2023;12:2301612. 10.1002/adhm.202301612 37552211

[30] Wang M, Wang J, Zhou P, Deng J, Zhao Y, Sun Y, Yang W, Wang D, Li Z, Hu X, et al. Nanoribbons self-assembled from short peptides demonstrate the formation of polar zippers between β-sheets. Nat Commun. 2018;9(1):5118. 30504813 10.1038/s41467-018-07583-2 PMC6269506

[31] Tao K, Xue B, Li Q, Hu W, Shimon LJW, Makam P, Si M, Yan X, Zhang M, Cao Y, et al. Stable and optoelectronic dipeptide assemblies for power harvesting. Mater Today. 2019;30:10–16. 10.1016/j.mattod.2019.04.002 PMC6850901 31719792

[32] Wang W, Wang L, Chen H, Zang J, Zhao X, Zhao G, Wang H. Selective elimination of the key subunit interfaces facilitates conversion of native 24-mer protein nanocage into 8-mer nanorings. J Am Chem Soc. 2018;140(43):14078–14081. 30336004 10.1021/jacs.8b09760

[33] Chen G, Huang S, Shen Y, Kou X, Ma X, Huang S, Tong Q, Ma K, Chen W, Wang P, et al. Protein-directed, hydrogen-bonded biohybrid framework. Chem. 2021;7(10):2722–2742.

[34] Zou Q, Abbas M, Zhao L, Li S, Shen G, Yan X. Biological photothermal nanodots based on self-assembly of peptide–porphyrin conjugates for antitumor therapy. J Am Chem Soc. 2017;139(5):1921–1927. 28103663 10.1021/jacs.6b11382

[35] Ma Q, Lei H, Cao Y. Intramolecular covalent bonds in gram-positive bacterial surface proteins. Chembiochem. 2022;23(20): Article e20220031. 10.1002/cbic.202200316 35801833

[36] Yuan JX, Zou XL, Qin Y, Liu T, Du GL, Luo B, Chi MC, Liu YH, Shao YZ, Zhao JM, et al. Anti-freeze, anti-dehydrating and stretchable triboelectric materials enabled by covalent-like hydrogen bond interaction. Nano Energy. 2024;131: Article 110215.

[37] Choudhury P, Das K, Das PK. l-Phenylalanine-tethered, naphthalene diimide-based, aggregation-induced, green-emitting organic nanoparticles. Langmuir. 2017;33(18):4500–4510. 28438019 10.1021/acs.langmuir.7b00452

[38] Liu K, Xing R, Chen C, Shen G, Yan L, Zou Q, Ma G, Möhwald H, Yan X. Peptide-induced hierarchical long-range order and photocatalytic activity of porphyrin assemblies. Angew Chem Int Ed Engl. 2015;54(2):500–505. 25377526 10.1002/anie.201409149

[39] Du M, Zhou K, Yu R, Zhai Y, Chen G, Wang Q. Noncovalent self-assembly of protein crystals with tunable structures. Nano Lett. 2021;21(4):1749–1757. 33556245 10.1021/acs.nanolett.0c04587

[40] Mandal S, Arief I, Chae S, Tahir M, Hoang TX, Heinrich G, Wießner S, Das A. Self-repairable hybrid piezoresistive-triboelectric sensor cum nanogenerator utilizing dual-dynamic reversible network in mechanically robust modified natural rubber. Adv Sensor Res. 2024;3(10):2400036.

[41] Gazit E. Self-assembled peptide nanostructures: The design of molecular building blocks and their technological utilization. Chem Soc Rev. 2007;36(8):1263–1269. 17619686 10.1039/b605536m

[42] Criado-Gonzalez M, Wagner D, Rodon Fores J, Blanck C, Schmutz M, Chaumont A, Rabineau M, Schlenoff JB, Fleith G, Combet J, et al. Supramolecular hydrogel induced by electrostatic interactions between polycation and phosphorylated-Fmoc-tripeptide. Chem Mater. 2020;32(5):1946–1956.

[43] Bai Y, Luo Q, Liu J. Protein self-assembly via supramolecular strategies. Chem Soc Rev. 2016;45(10):2756–2767. 27080059 10.1039/c6cs00004e

[44] Kostiainen MA, Hiekkataipale P, Laiho A, Lemieux V, Seitsonen J, Ruokolainen J, Ceci P. Electrostatic assembly of binary nanoparticle superlattices using protein cages. Nat Nanotechnol. 2013;8(1):52–56. 23241655 10.1038/nnano.2012.220

[45] Cheedarala RK, Parvez AN, Ahn KK. Electric impulse spring-assisted contact separation mode triboelectric nanogenerator fabricated from polyaniline emeraldine salt and woven carbon fibers. Nano Energy. 2018;53:362–372.

[46] Levin A, Hakala TA, Schnaider L, Bernardes GJL, Gazit E, Knowles TPJ. Biomimetic peptide self-assembly for functional materials. Nat Rev Chem. 2020;4(11):615–634. 39650726 10.1038/s41570-020-0215-y PMC7617017

[47] Liao H-S, Lin J, Liu Y, Huang P, Jin A, Chen X. Self-assembly mechanisms of nanofibers from peptide amphiphiles in solution and on substrate surfaces. Nanoscale. 2016;8(31):14814–14820. 27447093 10.1039/c6nr04672j PMC5226416

[48] Luo X, Huo Q, Liu X, Zheng C, Liu Y. Effect of hydrophilic or hydrophobic interactions on the self-assembly behavior and micro-morphology of a collagen mimetic peptide. J Leather Sci Eng. 2021;3(1):11.

[49] Jiao C, Li C, Yue J, Li L, Yang H, Tao Y, Lu J, Lv Y, Wang H, Tan M, et al. Structurally robust cellulosic triboelectric materials under high moisture conditions for self-powered sensing. Nano Energy. 2024;122: Article 109311.

[50] Lin M, Liu D, Gong Y, Shu L, Wang H, Zhang G, Li J, Gao Z, Sun J, Chen X. Bioactive assembly cofactor-assisted ursolic acid helix for enhanced anticancer efficacy via in situ virus-like transition. J Am Chem Soc. 2025;147(20):17010–17021. 40354555 10.1021/jacs.5c01214

[51] Bader T, Boone K, Johnson C, Berrie CL, Tamerler C. Probing solid-binding peptide self-assembly kinetics using a frequency response cooperativity model. Biomimetics. 2025;10(2):107. 39997130 10.3390/biomimetics10020107 PMC11853711

[52] William M, Jonathan B, Hastings G. Handbook of biomaterial properties. New York (NY): Springer; 2016.

[53] Han F, Zhu C, Guo Q, Yang H, Li B. Cellular modulation by the elasticity of biomaterials. J Mater Chem B. 2016;4(1):9–26. 32262805 10.1039/c5tb02077h

[54] Harley R, James D, Miller A, White JW. Phonons and the elastic moduli of collagen and muscle. Nature. 1977;267(5608):285–287. 865624 10.1038/267285a0

[55] Park J. Bioceramics: Properties, characterizations, and applications Springer; 2008.

[56] Xin X, Borzacchiello A, Netti PA, Ambrosio L, Nicolais L. Hyaluronic-acid-based semi-interpenetrating materials. J Biomater Sci Polym Ed. 2004;15:1223–1236. 15503636 10.1163/1568562041753025

[57] Bauer A, Gu L, Kwee B, Li WA, Dellacherie M, Celiz AD, Mooney DJ. Hydrogel substrate stress-relaxation regulates the spreading and proliferation of mouse myoblasts. Acta Biomater. 2017;62:82–90. 28864249 10.1016/j.actbio.2017.08.041 PMC5641979

[58] Wu Z, Yang Z, Sha D, Ma Y, Kim BYS, Jiang W, Yuan Y, Liu C. Injectable, viscoelastic hydrogel precisely regulates developmental tissue regeneration. Chem Eng J. 2022;434: Article 133860.

[59] Yang Z, Xue J, Shi Z, Zhang H, Yu X, Du L, Zhu Y, Huan Z, Wu C. Naturally derived flexible bioceramics: Biomass recycling approach and advanced function. Matter. 2024;7(3):1275–1291.

[60] Basu B. Mechanical properties of biomaterials. In:*Biomaterials for musculoskeletal regeneration: Concepts*. Berlin (Germany): Springer; 2016. p. 175–222.

[61] Launey ME, Ritchie RO. On the fracture toughness of advanced materials. Adv Mater. 2009;21(20):2103–2110.

[62] Hu Y, Li D, Wu L, Yang J, Jian X, Bin Y. Carbon nanotube buckypaper and buckypaper/polypropylene composites for high shielding effectiveness and absorption-dominated shielding material. Compos Sci Technol. 2019;181: Article 107699.

[63] Li J, Carlos C, Zhou H, Sui J, Wang Y, Silva-Pedraza Z, Yang F, Dong Y, Zhang Z, Hacker TA, et al. Stretchable piezoelectric biocrystal thin films. Nat Commun. 2023;14(1):6562. 37848410 10.1038/s41467-023-42184-8 PMC10582159

[64] Long Q, Jiang G, Zhou J, Zhao D, Yu H. A cellulose Ionogel with rubber-like stretchability for low-grade heat harvesting. Research. 2024;7:0533. 39559347 10.34133/research.0533 PMC11570788

[65] Deng W, Zhou Y, Libanori A, Chen G, Yang W, Chen J. Piezoelectric nanogenerators for personalized healthcare. Chem Soc Rev. 2022;51(9):3380–3435. 35352069 10.1039/d1cs00858g

[66] Guerin S, Tofail SAM, Thompson D. Organic piezoelectric materials: Milestones and potential. NPG Asia Mater. 2019;11(1):10.

[67] Perlovich GL, Hansen LK, Bauer-Brandl A. The polymorphism of glycine. Thermochemical and structural aspects. J Therm Anal Calorim. 2001;66(3):699–715.

[68] Guerin S, Stapleton A, Chovan D, Mouras R, Gleeson M, McKeown C, Noor MR, Silien C, Rhen FMF, Kholkin AL, et al. Control of piezoelectricity in amino acids by supramolecular packing. Nat Mater. 2018;17(2):180–186. 29200197 10.1038/nmat5045

[69] Hamley IW. Peptide nanotubes. Angew Chem Int Ed Engl. 2014;53(27):6866–6881. 24920517 10.1002/anie.201310006

[70] Lee J-H, Heo K, Schulz-Schönhagen K, Lee JH, Desai MS, Jin H-E, Lee S-W. Diphenylalanine peptide nanotube energy harvesters. ACS Nano. 2018;12(8):8138–8144. 30071165 10.1021/acsnano.8b03118

[71] Jenkins K, Kelly S, Nguyen V, Wu Y, Yang R. Piezoelectric diphenylalanine peptide for greatly improved flexible nanogenerators. Nano Energy. 2018;51:317–323.

[72] Kholkin A, Amdursky N, Bdikin I, Gazit E, Rosenman G. Strong piezoelectricity in bioinspired peptide nanotubes. ACS Nano. 2010;4(2):610–614. 20131852 10.1021/nn901327v

[73] Wang R, Sui J, Wang X. Natural piezoelectric biomaterials: A biocompatible and sustainable building block for biomedical devices. ACS Nano. 2022;16(11):17708–17728. 36354375 10.1021/acsnano.2c08164 PMC10040090

[74] Vijayakanth T, Liptrot DJ, Gazit E, Boomishankar R, Bowen CR. Recent advances in organic and organic-inorganic hybrid materials for piezoelectric mechanical energy harvesting. Adv Funct Mater. 2022;32(17):2109492.

[75] Fukada E. On the piezoelectric effect of silk fibers. J Phys Soc Jpn. 1956;11(12):1301A.

[76] Karan SK, Maiti S, Kwon O, Paria S, Maitra A, Si SK, Kim Y, Kim JK, Khatua BB. Nature driven spider silk as high energy conversion efficient bio-piezoelectric nanogenerator. Nano Energy. 2018;49:655–666.

[77] Zelenovskii PS, Vasileva DS, Vasilev SG, Kopyl S, Kholkin A. Ferroelectricity in glycine: A mini-review. Front Mater. 2022;9: Article 918890.

[78] Kontturi E, Laaksonen P, Linder MB, Nonappa, Groschel AH, Rojas OJ, Ikkala O. Advanced materials through assembly of nanocelluloses. Adv Mater. 2018;30(24):1703779. 10.1002/adma.201703779 29504161

[79] Yam S-C, Zain SM, Sanghiran Lee V, Chew K-H. Correlation between polar surface area and bioferroelectricity in DNA and RNA nucleobases. Eur Phys J E. 2018;41(7):86. 30014219 10.1140/epje/i2018-11696-5

[80] Chang R, Zhao L, Xing R, Li J, Yan X. Functional chromopeptide nanoarchitectonics: Molecular design, self-assembly and biological applications. Chem Soc Rev. 2023;52(8):2688–2712. 36987746 10.1039/d2cs00675h

[81] Zornberg LZ, Lewis DJ, Mertiri A, Hueckel T, Carter DJD, Macfarlane RJ. Self-assembling systems for optical out-of-plane coupling devices. ACS Nano. 2023;17(4):3394–3400. 36752596 10.1021/acsnano.2c08344

[82] Wang L, Li L-L, Ma HL, Wang H. Recent advances in biocompatible supramolecular assemblies for biomolecular detection and delivery. Chin Chem Lett. 2013;24(5):351–358.

[83] Fatma B, Andrabi SM, Gupta S, Verma V, Kumar A, Pitsalidis C, Garg A. Biocompatible, breathable and degradable microbial cellulose based triboelectric nanogenerator for wearable transient electronics. Nano Energy. 2023;114: Article 108628.

[84] Hu Y, Wang ZL. Recent progress in piezoelectric nanogenerators as a sustainable power source in self-powered systems and active sensors. Nano Energy. 2015;14:3–14.

[85] Hwang G-T, Kim Y, Lee J-H, Oh S, Jeong CK, Park DY, Ryu J, Kwon H, Lee S-G, Joung B, et al. Self-powered deep brain stimulation via a flexible PIMNT energy harvester. Energy Environ Sci. 2015;8(9):2677–2684.

[86] Wang ZL, Song J. Piezoelectric nanogenerators based on zinc oxide nanowire arrays. Science. 2006;312(5771):242–246. 16614215 10.1126/science.1124005

[87] Yang R, Qin Y, Li C, Zhu G, Wang ZL. Converting biomechanical energy into electricity by a muscle-movement-driven nanogenerator. Nano Lett. 2009;9(3):1201–1205. 19203203 10.1021/nl803904b

[88] Huang C-T, Song J, Lee W-F, Ding Y, Gao Z, Hao Y, Chen L-J, Wang ZL. GaN nanowire arrays for high-output nanogenerators. J Am Chem Soc. 2010;132(13):4766–4771. 20218713 10.1021/ja909863a

[89] Lin L, Lai C-H, Hu Y, Zhang Y, Wang X, Xu C, Snyder RL, Chen L-J, Wang ZL. High output nanogenerator based on assembly of GaN nanowires. Nanotechnology. 2011;22(47): Article 475401. 22048156 10.1088/0957-4484/22/47/475401

[90] Jung W-S, Lee M-J, Kang M-G, Moon HG, Yoon S-J, Baek S-H, Kang C-Y. Powerful curved piezoelectric generator for wearable applications. Nano Energy. 2015;13:174–181.

[91] Pi Z, Zhang J, Wen C, Zhang Z-B, Wu D. Flexible piezoelectric nanogenerator made of poly(vinylidenefluoride-co-trifluoroethylene) (PVDF-TrFE) thin film. Nano Energy. 2014;7:33–41.

[92] Chen X, Tian H, Li X, Shao J, Ding Y, An N, Zhou Y. A high performance P(VDF-TrFE) nanogenerator with self-connected and vertically integrated fibers by patterned EHD pulling. Nanoscale. 2015;7(27):11536–11544. 25981294 10.1039/c5nr01746g

[93] Chen S, Tong X, Huo Y, Liu S, Yin Y, Tan M-L, Cai K, Ji W. Piezoelectric biomaterials inspired by nature for applications in biomedicine and nanotechnology. Adv Mater. 2024;36(35):2406192. 10.1002/adma.202406192 39003609

[94] Niu Q, Wei H, Hsiao BS, Zhang Y. Biodegradable silk fibroin-based bio-piezoelectric/triboelectric nanogenerators as self-powered electronic devices. Nano Energy. 2022;96: Article 107101.

[95] Vu N, Jenkins K, Yang R. Epitaxial growth of vertically aligned piezoelectric diphenylalanine peptide microrods with uniform polarization. Nano Energy. 2015;17:323–329.

[96] Nguyen V, Zhu R, Jenkins K, Yang R. Self-assembly of diphenylalanine peptide with controlled polarization for power generation. Nat Commun. 2016;7(1):13566. 27857133 10.1038/ncomms13566 PMC5120215

[97] Iitaka Y. A new form of glycine. Proc Japan Acad. 1954;30(2):109–112.

[98] Yang F, Li J, Long Y, Zhang Z, Wang L, Sui J, Dong Y, Wang Y, Taylor R, Ni D, et al. Wafer-scale heterostructured piezoelectric bio-organic thin films. Science. 2021;373(6552):337–342. 34437153 10.1126/science.abf2155 PMC8516594

[99] Wang Y, Liu S, Li L, Li H, Yin Y, Rencus-Lazar S, Guerin S, Ouyang W, Thompson D, Yang R, et al. Manipulating the piezoelectric response of amino acid-based assemblies by supramolecular engineering. J Am Chem Soc. 2023;145(28):15331–15342. 37392396 10.1021/jacs.3c02993

[100] Sencadas V, Garvey C, Mudie S, Kirkensgaard JJK, Gouadec G, Hauser S. Electroactive properties of electrospun silk fibroin for energy harvesting applications. Nano Energy. 2019;66: Article 104106.

[101] Stapleton A, Noor MR, Sweeney J, Casey V, Kholkin AL, Silien C, Gandhi AA, Soulimane T, Tofail SAM. The direct piezoelectric effect in the globular protein lysozyme. Appl Phys Lett. 2017;111(14): Article 142902.

[102] Bdikin I, Heredia A, Neumayer SM, Bystrov VS, Gracio J, Rodriguez BJ, Kholkin AL. Local piezoresponse and polarization switching in nucleobase thymine microcrystals. J Appl Phys. 2015;118(7): Article 072007.

[103] Lee BY, Zhang J, Zueger C, Chung W-J, Yoo SY, Wang E, Meyer J, Ramesh R, Lee S-W. Virus-based piezoelectric energy generation. Nat Nanotechnol. 2012;7(6):351–356. 22581406 10.1038/nnano.2012.69

[104] Das R, Curry EJ, Le TT, Awale G, Liu Y, Li S, Contreras J, Bednarz C, Millender J, Xin X, et al. Biodegradable nanofiber bone-tissue scaffold as remotely-controlled and self-powering electrical stimulator. Nano Energy. 2020;76: Article 105028. 38074984 10.1016/j.nanoen.2020.105028 PMC10703347

[105] Ouyang H, Liu Z, Li N, Shi B, Zou Y, Xie F, Ma Y, Li Z, Li H, Zheng Q, et al. Symbiotic cardiac pacemaker. Nat Commun. 2019;10(1):1821. 31015519 10.1038/s41467-019-09851-1 PMC6478903

[106] Shi R, Zhang J, Tian J, Zhao C, Li Z, Zhang Y, Li Y, Wu C, Tian W, Li Z. An effective self-powered strategy to endow titanium implant surface with associated activity of anti-biofilm and osteogenesis. Nano Energy. 2020;77: Article 105201.

[107] Wang J, Li S, Yi F, Zi Y, Lin J, Wang X, Xu Y, Wang ZL. Sustainably powering wearable electronics solely by biomechanical energy. Nat Commun. 2016;7(1):12744. 27677971 10.1038/ncomms12744 PMC5052715

[108] Tao Z, Yuan H, Ding S, Wang Y, Hu W, Yang R. Diphenylalanine-based degradable piezoelectric nanogenerators enabled by polylactic acid polymer-assisted transfer. Nano Energy. 2021;88: Article 106229.

[109] Patil SR, Chougale MY, Kim J, Shaukat RA, Noman M, Saqib QM, Patil CS, Ghode SB, Dongale TD, Dubal D, et al. Nature-driven edible black soldier Fly(BSF) insect larvae derived chitin biofilm for sustainable multifunctional energy harvesting. Adv Sustain Syst. 2024;8(2):2300312.

[110] Sun Z, Wang S, Zhao S, Wei H, Shen G, Pu Y, Zhang S. Achieving low energy consuming bio-based piezoelectric nanogenerators via modulating the inner layer thickness for a highly sensitive pedometer. J Mater Chem C. 2024;12(3):859–867.

[111] Hu J, Liu S, Huo Y, Yang B, Yin Y, Tan M-L, Liu P, Cai K, Ji W. Piezoelectric vitamin-based self-assemblies for energy generation. Adv Mater. 2025;37(9):2417409. 10.1002/adma.202417409 39838767

[112] Wu C, Wang AC, Ding W, Guo H, Wang ZL. Triboelectric nanogenerator: A foundation of the energy for the new era. Adv Energy Mater. 2019;9(1):1802906.

[113] Huang J, Jiang T, Zhang Z, Zhang W, Wang S, Chen Z, Wan J, Li P, Li H, Gui C. Fabrication of biomaterial-based triboelectric nanogenerators: Study of the relationship between output performance and strain in dielectric materials. ACS Sustain Chem Eng. 2023;11(26):9540–9552.

[114] Li Y, Liu X, Ren Z, Luo J, Zhang C, Cao C, Yuan H, Pang Y. Marine biomaterial-based triboelectric nanogenerators: Insights and applications. Nano Energy. 2024;119: Article 109046.

[115] Yuan H, Zhang J, Rencus-Lazar S, Ren Z, Lin R, Gazit E, Yang R. The engineering of molecular packing in amino acid crystals for the enhanced triboelectric effect. Nano Energy. 2023;110: Article 108375.

[116] Park IW, Choi J, Kim KY, Jeong J, Gwak D, Lee Y, Ahn YH, Choi YJ, Hong YJ, Chung W-J, et al. Vertically aligned cyclo-phenylalanine peptide nanowire-based high-performance triboelectric energy generator. Nano Energy. 2019;57:737–745.

[117] Xiong W, Hu K, Li Z, Jiang Y, Li Z, Li Z, Wang X. A wearable system based on core-shell structured peptide-Co_9_S_8_ supercapacitor and triboelectric nanogenerator. Nano Energy. 2019;66: Article 104149.

[118] Zhang Y, Zhou Z, Sun L, Liu Z, Xia X, Tao TH. “Genetically engineered” biofunctional triboelectric nanogenerators using recombinant spider silk. Adv Mater. 2018;30(50):1805722. 10.1002/adma.201805722 30306646

[119] Tan X, Huang Z, Pei H, Jia Z, Zheng J. Highly porous, ultralight, biocompatible silk fibroin aerogel-based triboelectric nanogenerator. ACS Sens. 2024;9(8):3938–3946. 39096301 10.1021/acssensors.4c00401

[120] Panda S, Hajra S, Kim H-G, Achary PGR, Pakawanit P, Yang Y, Mishra YK, Kim HJ. Sustainable solutions for oral health monitoring: Biowaste-derived triboelectric nanogenerator. ACS Appl Mater Interfaces. 2023;15(30):36096–36106. 37471608 10.1021/acsami.3c04024

[121] Jiang W, Li H, Liu Z, Li Z, Tian J, Shi B, Zou Y, Ouyang H, Zhao C, Zhao L, et al. Fully bioabsorbable natural-materials-based triboelectric nanogenerators. Adv Mater. 2018;30(32):1801895. 10.1002/adma.201801895 29947102

[122] Dong K, Wu Z, Deng J, Wang AC, Zou H, Chen C, Hu D, Gu B, Sun B, Wang ZL. A stretchable yarn embedded triboelectric nanogenerator as electronic skin for biomechanical energy harvesting and multifunctional pressure sensing. Adv Mater. 2018;30(43):1804944. 10.1002/adma.201804944 30256476

[123] Yin J, Li X, Yu J, Zhang Z, Zhou J, Guo W. Generating electricity by moving a droplet of ionic liquid along graphene. Nat Nanotechnol. 2014;9(5):378–383. 24705513 10.1038/nnano.2014.56

[124] Yang S, Su Y, Xu Y, Wu Q, Zhang Y, Raschke MB, Ren M, Chen Y, Wang J, Guo W, et al. Mechanism of electric power generation from ionic droplet motion on polymer supported graphene. J Am Chem Soc. 2018;140(42):13746–13752. 30257558 10.1021/jacs.8b07778

[125] Zhou X, Zhang W, Zhang C, Tan Y, Guo J, Sun Z, Deng X. Harvesting electricity from water evaporation through microchannels of natural wood. ACS Appl Mater Interfaces. 2020;12(9):11232–11239. 32048827 10.1021/acsami.9b23380

[126] Das SS, Pedireddi VM, Bandopadhyay A, Saha P, Chakraborty S. Electrical power generation from wet textile mediated by spontaneous nanoscale evaporation. Nano Lett. 2019;19(10):7191–7200. 31507187 10.1021/acs.nanolett.9b02783

[127] Yun TG, Bae J, Rothschild A, Kim I-D. Transpiration driven electrokinetic power generator. ACS Nano. 2019;13(11):12703–12709. 31618009 10.1021/acsnano.9b04375

[128] Chen X, Goodnight D, Gao Z, Cavusoglu AH, Sabharwal N, DeLay M, Driks A, Sahin O. Scaling up nanoscale water-driven energy conversion into evaporation-driven engines and generators. Nat Commun. 2015;6(1):7346. 26079632 10.1038/ncomms8346 PMC4490384

[129] Liu X, Ueki T, Gao H, Woodard TL, Nevin KP, Fu T, Fu S, Sun L, Lovley DR, Yao J. Microbial biofilms for electricity generation from water evaporation and power to wearables. Nat Commun. 2022;13(1):4369. 35902587 10.1038/s41467-022-32105-6 PMC9334603

[130] Xiao P, He J, Ni F, Zhang C, Liang Y, Zhou W, Gu J, Xia J, Kuo S-W, Chen T. Exploring interface confined water flow and evaporation enables solar-thermal-electro integration towards clean water and electricity harvest via asymmetric functionalization strategy. Nano Energy. 2020;68: Article 104385.

[131] Venkateshaiah A, Cheong JY, Shin S-H, Akshaykumar KP, Yun TG, Bae J, Wacławek S, Černík M, Agarwal S, Greiner A, et al. Recycling non-food-grade tree gum wastes into nanoporous carbon for sustainable energy harvesting. Green Chem. 2020;22(4):1198–1208.

[132] Miettunen K, Hadadian M, Garcia JV, Lawrynowicz A, Akulenko E, Rojas OJ, Hummel M, Vapaavuori J. Bio-based materials for solar cells. WIRES Energy Environ. 2024;13(1): Article e508.

[133] Han J, Tian Y, Jeon I. Natural and nature-inspired biomaterial additives for metal halide perovskite optoelectronics. Adv Mater. 2025;37(26):2410327. 10.1002/adma.202410327 39523718

[134] Liu B, Ren X, Li R, Chen Y, He D, Li Y, Zhou Q, Ma D, Han X, Shai X, et al. Stabilizing top interface by molecular locking strategy with polydentate chelating biomaterials toward efficient and stable perovskite solar cells in ambient air. Adv Mater. 2024;36(19):2312679. 10.1002/adma.202312679 38300149

[135] Li J, Wang N, Wang Y, Liang Z, Peng Y, Yang C, Bao X, Xia Y. Efficient inverted organic solar cells with a thin natural biomaterial L-arginine as electron transport layer. Sol Energy. 2020;196:168–176.

[136] Zhang P, Gu N, Song L, Chen W-H, Du P, Yin X, Xiong J. Bifunctional green cellulose derivatives employed for high efficiency and stable perovskite solar cells under ambient environment. J Alloys Compd. 2021;886: Article 161247.

[137] Li Y, Cheng M, Jungstedt E, Xu B, Sun L, Berglund L. Optically transparent wood substrate for perovskite solar cells. ACS Sustain Chem Eng. 2019;7(6):6061–6067. 30918764 10.1021/acssuschemeng.8b06248 PMC6430497

[138] Tiihonen A, Siipola V, Lahtinen K, Pajari H, Widsten P, Tamminen T, Kallio T, Miettunen K. Biocarbon from brewery residues as a counter electrode catalyst in dye solar cells. Electrochim Acta. 2021;368: Article 137583.

[139] Gao L, Zhou Y, Meng F, Li Y, Liu A, Li Y, Zhang C, Fan M, Wei G, Ma T. Several economical and eco-friendly bio-carbon electrodes for highly efficient perovskite solar cells. Carbon. 2020;162:267–272.

[140] Sharma A, Singh G, Arya SK. Biofuel cell nanodevices. Int J Hydrog Energy. 2021;46(4):3270–3288.

[141] Dong T, Knoshaug EP, Pienkos PT, Laurens LML. Lipid recovery from wet oleaginous microbial biomass for biofuel production: A critical review. Appl Energy. 2016;177:879–895.

[142] Guo Y, Chen C, Feng J, Wang L, Wang J, Tang C, Sun X, Peng H. An anti-biofouling flexible fiber biofuel cell working in the brain. Small Methods. 2022;6(5):2200142. 10.1002/smtd.202200142 35322598

[143] Kavaliauskait G, Virbickas P, Ziziunaite G, Ramanavicius A, Valiuniene A. Biofuel cell based on yeast modified with Prussian blue. J Electroanal Chem. 2023;928: Article 117079.

[144] Raheem A, Prinsen P, Vuppaladadiyam AK, Zhao M, Luque R. A review on sustainable microalgae based biofuel and bioenergy production: Recent developments. J Clean Prod. 2018;181:42–59.

[145] Lee J, Kim S, You S, Park Y-K. Bioenergy generation from thermochemical conversion of lignocellulosic biomass-based integrated renewable energy systems. Renew Sust Energy Rev. 2023;178: Article 113240.

[146] Khan F, Kim DH, Lee J. Functionalized materials and geometric designs of thermoelectric devices for smart wearable applications. Appl Energy. 2025;379: Article 124940.

[147] Jia S, Ma H, Gao S, Yang L, Sun Q. Thermoelectric materials and devices for advanced biomedical applications. Small. 2024;20(51):2405019. 10.1002/smll.202405019 39392147

[148] Sun H, Tang F, Bi Y, Sun H, Huang L, Jiang F, Chen L, Li J. Hierarchically porous cellulose membrane via self-assembly engineering for ultra high-power thermoelectrical generation in natural convection. Adv Funct Mater. 2023;33(52):2307960.

[149] Chen Y, Zhang H, Hong G, Li L, Qu Q. Human bio-electric generator: Self-powered cellulose-based wearable sensor with ultra-stretchability and low-grade body heat harvesting. Carbohydr Polym. 2025;355: Article 123349. 40037729 10.1016/j.carbpol.2025.123349

[150] Wu T, Xu W, Li X, Du Y, Sheng M, Zhong H, Xie H, Qu J. Bioinspired micro/nanostructured polyethylene/poly(ethylene oxide)/graphene films with robust superhydrophobicity and excellent antireflectivity for solar-thermal power generation, thermal management, and afterheat utilization. ACS Nano. 2022;16(10):16624–16635. 36240110 10.1021/acsnano.2c06065

[151] Yan J, Zhang X, Zhu N, Qin Y, Yang G. High-performance triboelectric nanogenerators boosted by synergistically aligned piezoelectric/conductive composite nanofibers. Polym Compos. 2025;46(4):3228–3238.

[152] Zhao C, Zhang Q, Zhang W, Du X, Zhang Y, Gong S, Ren K, Sun Q, Wang ZL. Hybrid piezo/triboelectric nanogenerator for highly efficient and stable rotation energy harvesting. Nano Energy. 2019;57:440–449.

[153] Hussain SZ, Singh VP, Sadeque MSB, Yavari S, Kalimuldina G, Ordu M. Piezoelectric-triboelectric hybrid nanogenerator for energy harvesting and self-powered sensing applications. Small. 2025;21(43): Article e2504626. 40708341 10.1002/smll.202504626 PMC12571219

[154] Rozbeský D, Rosůlek M, Kukačka Z, Chmelík J, Man P, Novák P. Impact of chemical cross-linking on protein structure and function. Anal Chem. 2018;90(2):1104–1113. 29232109 10.1021/acs.analchem.7b02863

[155] Park S, Lee K-S, Bozoklu G, Cai W, Nguyen ST, Ruoff RS. Graphene oxide papers modified by divalent ions—Enhancing mechanical properties via chemical cross-linking. ACS Nano. 2008;2(3):572–578. 19206584 10.1021/nn700349a

[156] Ding S, Zhai H, Tao X, Yang P, Liu Z, Qin S, Hong Z, Chen X, Wang ZL. A triboelectric-electromagnetic hybrid nanogenerator with magnetic coupling assisted waterproof encapsulation for long-lasting energy harvesting. Small. 2024;20(42):2403879. 10.1002/smll.202403879 38881274

[157] Dong R, Liu Y, Mou L, Deng J, Jiang X. Microfluidics-based biomaterials and biodevices. Adv Mater. 2019;31(45):1805033. 10.1002/adma.201805033 30345586

[158] Chen Z, Lv Z, Zhang Z, Zhang Y, Cui W. Biomaterials for microfluidic technology. Mater. Futures. 2022;1(1): Article 012401.

[159] McDonald SM, Augustine EK, Lanners Q, Rudin C, Catherine Brinson L, Becker ML. Applied machine learning as a driver for polymeric biomaterials design. Nat Commun. 2023;14(1):4838. 37563117 10.1038/s41467-023-40459-8 PMC10415291

[160] Patel S, Dwivedi A, Darwhekar GN. AI-driven advancements in biomaterials science: A narrative review. Premier J Sci. 2025;12: Article 100092.

[161] Fleming S, Ulijn RV. Design of nanostructures based on aromatic peptide amphiphiles. Chem Soc Rev. 2014;43(23):8150–8177. 25199102 10.1039/c4cs00247d

[162] Kim NH, Choi H, Shahzad ZM, Ki H, Lee J, Chae H, Kim YH. Supramolecular assembly of protein building blocks: From folding to function. Nano Converg. 2022;9(1):4–20. 35024976 10.1186/s40580-021-00294-3 PMC8755899

[163] Hosseini ES, Manjakkal L, Dahiya R. Bio-organic glycine based flexible piezoelectric stress sensor for wound monitoring. In: *2018 IEEE Sensors*. New Delhi (India): IEEE; 2018. p. 1–4.

[164] Hosseini ES, Manjakkal L, Shakthivel D, Dahiya R. Glycine–chitosan-based flexible biodegradable piezoelectric pressure sensor. ACS Appl Mater Interfaces. 2020;12(8):9008–9016. 32011853 10.1021/acsami.9b21052 PMC7146751

[165] Hoque NA, Thakur P, Biswas P, Saikh MM, Roy S, Bagchi B, Das S, Ray PP. Biowaste crab shell-extracted chitin nanofiber-based superior piezoelectric nanogenerator. J Mater Chem A. 2018;6(28):13848–13858.

[166] Shin D-M, Han HJ, Kim W-G, Kim E, Kim C, Hong SW, Kim HK, Oh J-W, Hwang Y-H. Bioinspired piezoelectric nanogenerators based on vertically aligned phage nanopillars. Energy Environ Sci. 2015;8(11):3198–3203.

[167] Ghosh SK, Mandal D. High-performance bio-piezoelectric nanogenerator made with fish scale. Appl Phys Lett. 2016;109(10): Article 103701.

[168] Ghosh SK, Mandal D. Efficient natural piezoelectric nanogenerator: Electricity generation from fish swim bladder. Nano Energy. 2016;28:356–365.

[169] Ghosh SK, Mandal D. Bio-assembled, piezoelectric prawn shell made self-powered wearable sensor for non-invasive physiological signal monitoring. Appl Phys Lett. 2017;110(12): Article 123701.

[170] Maiti S, Karan SK, Lee J, Mishra AK, Khatua BB, Kim JK. Bio-waste onion skin as an innovative nature-driven piezoelectric material with high energy conversion efficiency. Nano Energy. 2017;42:282–293.

[171] Vivekananthan V, Alluri NR, Purusothaman Y, Chandrasekhar A, Selvarajan S, Kim S-J. Biocompatible collagen nanofibrils: An approach for sustainable energy harvesting and battery-free humidity sensor applications. ACS Appl Mater Interfaces. 2018;10(22):18650–18656. 29742894 10.1021/acsami.8b02915

[172] Saqib QM, Khan MU, Song H, Chougale MY, Shaukat RA, Kim J, Bae J, Choi MJ, Kim SC, Kwon O, et al. Natural hierarchically structured highly porous tomato peel based tribo- and piezo-electric nanogenerator for efficient energy harvesting. Adv Sustain Syst. 2021;5(7):2100066.

[173] Bairagi S, Ghosh S, Ali SW. A fully sustainable, self-poled, bio-waste based piezoelectric nanogenerator: Electricity generation from pomelo fruit membrane. Sci Rep. 2020;10(1):12121. 32694668 10.1038/s41598-020-68751-3 PMC7374593

[174] Sun J, Guo H, Ribera J, Wu C, Tu K, Binelli M, Panzarasa G, Schwarze FWMR, Wang ZL, Burgert I. Sustainable and biodegradable wood sponge piezoelectric nanogenerator for sensing and energy harvesting applications. ACS Nano. 2020;14(11):14665–14674. 32936611 10.1021/acsnano.0c05493

[175] Karan SK, Maiti S, Paria S, Maitra A, Si SK, Kim JK, Khatua BB. A new insight towards eggshell membrane as high energy conversion efficient bio-piezoelectric energy harvester. Mater Today Energy. 2018;9:114–125.

[176] Kar E, Barman M, Das S, Das A, Datta P, Mukherjee S, Tavakoli M, Mukherjee N, Bose N. Chicken feather fiber-based bio-piezoelectric energy harvester: An efficient green energy source for flexible electronics. Sustain Energy Fuels. 2021;5(6):1857–1866.

[177] Shao D, Wang C, Li W, Lu L, Lu J, Yang W. Natural ginkgo tree leaves as piezo-energy harvesters. J Mater Chem C. 2022;10(40):15016–15027.

[178] Ghosal C, Ghosh SK, Roy K, Chattopadhyay B, Mandal D. Environmental bacteria engineered piezoelectric bio-organic energy harvester towards clinical applications. Nano Energy. 2022;93: Article 106843.

[179] Tiwari S, Devi A, Dubey DK, Maiti P. Induced piezoelectricity in cotton-based composites for energy-harvesting applications. ACS Appl Bio Mater. 2023;6(4):1536–1545. 10.1021/acsabm.2c01062 36848659

[180] Kar E, Ghosh P, Pratihar S, Tavakoli M, Sen S. Nature-driven biocompatible epidermal electronic skin for real-time wireless monitoring of human physiological signals. ACS Appl Mater Interfaces. 2023;15(16):20372–20384. 37067294 10.1021/acsami.3c00509

[181] Chougale MY, Khan MU, Kim J, Cosgrove J, Shaukat RA, Saqib QM, Banjade M, Patil SR, Brown C, Dubal D, et al. Snake ecdysis: A potential e-material for advanced electronic technology. Nano Energy. 2023;111: Article 108399.

[182] Kim M-S, Commerell W, Roh J-W, Park S-S. Degumming effects of silk fabrics as piezoelectrics for nanogenerators. Mater Sci Eng B Adv. 2023;298: Article 116898.

[183] Khan, M. U.; Mohammad, E.; Abbas, Y.; Rezeq, M. d.; Mohammad, B., Chicken skin based milli watt range biocompatible triboelectric nanogenerator for biomechanical energy harvesting. Sci Rep 2023, 13(1), 10160. 37349344 10.1038/s41598-023-36817-7 PMC10287749

[184] Lee M, Shin J, Kim S, Gandla S. Whey protein isolate film and laser-ablated textured PDMS-based single-electrode triboelectric nanogenerator for pressure-sensor application. Sensors. 2022;22(6):2154. 35336324 10.3390/s22062154 PMC8953929

[185] Luo H, Du J, Yang P, Shi Y, Liu Z, Yang D, Zheng L, Chen X, Wang ZL. Human-machine interaction via dual modes of voice and gesture enabled by triboelectric nanogenerator and machine learning. ACS Appl Mater Interfaces. 2023;15(13):17009–17018. 36947663 10.1021/acsami.3c00566 PMC10080540

[186] Bairagi S, Khandelwal G, Karagiorgis X, Gokhool S, Kumar C, Min G, Mulvihill DM. High-performance triboelectric nanogenerators based on commercial textiles: Electrospun nylon 66 nanofibers on silk and PVDF on polyester. ACS Appl Mater Interfaces. 2022;14(39):44591–44603. 36150147 10.1021/acsami.2c13092 PMC9542703

[187] Gong H, Xu Z, Yang Y, Xu Q, Li X, Cheng X, Huang Y, Zhang F, Zhao J, Li S, et al. Transparent, stretchable and degradable protein electronic skin for biomechanical energy scavenging and wireless sensing. Biosens Bioelectron. 2020;169: Article 112567. 32947084 10.1016/j.bios.2020.112567

[188] Li YT, Tian H, Zhao HM, Jian MQ, Lv YJ, Tian Y, Wang Q, Yang Y, Xiang Y, Zhang Y, et al. A novel cell-scale bio-nanogenerator based on electron-ion interaction for fast light power conversion. Nanoscale. 2018;10(2):526–532. 29255823 10.1039/c7nr07671a

[189] Lin MF, Chang PY, Lee CH, Wu XX, Jeng RJ, Chen CP. Biowaste eggshell membranes for bio-triboelectric nanogenerators and smart sensors. ACS Omega. 2023;8(7):6699–6707. 36844511 10.1021/acsomega.2c07292 PMC9948195

[190] Jing X, Li H, Mi HY, Feng PY, Tao X, Liu Y, Liu C, Shen C. Enhancing the performance of a stretchable and transparent triboelectric nanogenerator by optimizing the hydrogel ionic electrode property. ACS Appl Mater Interfaces. 2020;12(20):23474–23483. 32352755 10.1021/acsami.0c04219

[191] Joshi SR, Kim S. High power triboelectric nanogenerator based on nanofibers of silk protein and PVBVA and its motion sensing applications. Chem Eng J. 2024;489: Article 151248.

[192] Han Y, Han Y, Zhang X, Li L, Zhang C, Liu J, Lu G, Yu HD, Huang W. Fish gelatin based triboelectric nanogenerator for harvesting biomechanical energy and self-powered sensing of human physiological signals. ACS Appl Mater Interfaces. 2020;12(14):16442–16450. 32172560 10.1021/acsami.0c01061

[193] Liu X, Gao H, Ward JE, Liu X, Yin B, Fu T, Chen J, Lovley DR, Yao J. Power generation from ambient humidity using protein nanowires. Nature. 2020;578(7796):550–554. 32066937 10.1038/s41586-020-2010-9

[194] Gong F, Li H, Zhou Q, Wang M, Wang W, Lv Y, Xiao R, Papavassiliou DV. Agricultural waste-derived moisture-absorber for all-weather atmospheric water collection and electricity generation. Nano Energy. 2020;74: Article 104922.

[195] Lyu Q, Peng B, Xie Z, Du S, Zhang L, Zhu J. Moist-induced electricity generation by electrospun cellulose acetate membranes with optimized porous structures. ACS Appl Mater Interfaces. 2020;12(51):57373–57381. 33306344 10.1021/acsami.0c17931

[196] Mandal S, Mandal A, Jana G, Mallik S, Roy S, Ghosh A, Chattaraj PK, Goswami DK. Low operating voltage organic field-effect transistors with gelatin as a moisture-induced ionic dielectric layer: The issues of high carrier mobility. ACS Appl Mater Interfaces. 2020;12(17):19727–19736. 32233358 10.1021/acsami.0c01499

[197] Li M, Zong L, Yang W, Li X, You J, Wu X, Li Z, Li C. Biological nanofibrous generator for electricity harvest from moist air flow. Adv Funct Mater. 2019;29(32):1901798.

[198] Gao X, Xu T, Shao C, Han Y, Lu B, Zhang Z, Qu L. Electric power generation using paper materials. J Mater Chem A. 2019;7(36):20574–20578.

